# Microtubule dynamics are defined by conformations and stability of clustered protofilaments

**DOI:** 10.1073/pnas.2424263122

**Published:** 2025-05-29

**Authors:** Maksim Kalutskii, Helmut Grubmüller, Vladimir A. Volkov, Maxim Igaev

**Affiliations:** ^a^Department of Theoretical and Computational Biophysics, Max Planck Institute for Multidisciplinary Sciences, Göttingen D-37077, Germany; ^b^Centre for Molecular Cell Biology, School of Biological and Behavioural Sciences, Queen Mary University of London, London E1 4NS, United Kingdom; ^c^Division of Computational Biology, School of Life Sciences, University of Dundee, Dundee DD1 5EH, United Kingdom

**Keywords:** microtubule, dynamic instability, cryoelectron tomography, molecular dynamics simulation, coarse-grained modeling

## Abstract

Microtubules are dynamic cytoskeletal polymers that add and lose tubulin dimers at their ends. Microtubule growth, shortening, and transitions between them are linked to GTP hydrolysis. Recent evidence suggests that flexible tubulin protofilaments at microtubule ends adopt a variety of shapes, complicating structural analysis using conventional techniques. Therefore, the link between GTP hydrolysis, protofilament structure and microtubule polymerization state is poorly understood. Here, we investigate the conformational dynamics of microtubule ends using coarse-grained modeling supported by atomistic simulations and cryoelectron tomography. We show that individual bent protofilaments organize in clusters, transient precursors to the straight microtubule lattice, with GTP-bound ends showing elevated and more persistent cluster formation. Differences in the mechanical properties of GTP- and GDP-protofilaments result in differences in intracluster tension, determining both clustering propensity and protofilament length. We propose that conformational selection at microtubule ends favors long-lived clusters of short GTP-protofilaments that are more prone to forming a straight microtubule lattice and accommodating new tubulin dimers. Conversely, microtubule ends trapped in states with unevenly long and stiff GDP-protofilaments are more prone to shortening. We conclude that protofilament clustering is the key phenomenon that links the hydrolysis state of single tubulins to the polymerization state of the entire microtubule.

## Significance

Microtubules are essential components of the cytoskeleton with crucial roles in cell division and transport. Understanding microtubule growth and shortening is vital for obtaining insights into cellular function in normal and disease states. Microtubule ends adopt transient and heterogeneous shapes, making conventional approaches ineffective in solving their structures. This study combines biomolecular simulations and cryoelectron tomography to show how clustering of curved tubulin oligomers at microtubule ends determines whether a microtubule will elongate or shorten. We find that clustering is affected by the chemical state of tubulin proteins (GTP- or GDP-bound). This finding links the nucleotide hydrolysis events within individual tubulin proteins to the mechanical stability of intermediate clusters and, finally, to the polymerization state of the entire microtubule.

Microtubules are conserved cytoskeletal polymers which play crucial roles in processes ranging from cell division to neuronal homeostasis (1, 2). Moreover, microtubule assembly and disassembly can produce mechanical forces in the piconewton range (3–7). The remarkable property of microtubules to stochastically transition between phases of growth and shortening (8) has been linked to the binding and hydrolysis of GTP coupled to the addition of tubulin to microtubule ends (9). The idea that GTP is required for polymerization, while GTP hydrolysis upon polymerization renders microtubules intrinsically unstable is largely undisputed and is reflected in the concept of a stabilizing “GTP cap” (8). The GTP cap at the microtubule end is proposed to span hundreds of nanometers of microtubule length (10–12) and to maintain polymer stability, while its loss can trigger depolymerization.

While the GTP cap is a simple, yet fundamental concept for understanding tubulin polymerization, it does not explain the coupling between GTP hydrolysis and microtubule (dis)assembly, limiting predictions of microtubule behavior in various physiological and pathological contexts. The first models describing the mechanochemical cycle of tubulin (8, 13) were proposed before the crystal structure of tubulin was resolved (14, 15). Early electron microscopy studies revealed growing microtubule ends as mostly blunt or carrying sheet-like extensions, and shortening ones as flared, with protofilaments curling outward (16–19). Despite the fact that these studies relied on 2D projections and did not resolve 3D structures of microtubule ends explicitly, they elegantly explained why GDP-bound tubulin would not polymerize: Only GTP-bound tubulin adopts the necessary straight conformation to bond with neighboring tubulins. An extension of this model with three discrete states of tubulin, straight, mildly curved, and curved, was also proposed (20). However, structural characterization found no significant differences between the conformations of GTP- and GDP-bound tubulin in solution, (21, 22) in crystals, (23–25) and in silico, (26–29) challenging this hypothesis. Despite this controversy, the idea of nucleotide-dependent tubulin curvature has been incorporated into several minimal models of microtubule assembly (30–36).

Recent cryoelectron tomography (cryoET) work by McIntosh, Gudimchuk et al. (37, 38) and others (39) resolved 3D structures of both growing and shortening microtubule ends explicitly, and reported curled protofilaments in both polymerization states, in vitro as well as in vivo, implying profound changes to the tubulin polymerization paradigm. Moreover, cryoET reconstructions revealed that protofilaments were flexible in the radial plane of bending, whereas stepping out of that plane was limited to less than 10% of the protofilament length, suggesting a much higher tangential rigidity and no interactions between adjacent protofilaments (37, 38). Consequently, it was proposed that microtubule growth is achieved by thermal fluctuations of similarly curved and independent protofilaments, with only the bonds between GTP–tubulins being stable enough to maintain a straight lattice. This idea was further explored by the same authors using Brownian Dynamics modeling (37, 38, 40).

Most recently, we and others have performed large-scale atomistic simulations of complete GTP- and GDP-microtubule end models over the course of microseconds (41–43). In particular, our previous study (42) confirmed that all microtubule ends tend to be flared regardless of the nucleotide state; however, other key observations were incompatible with the original findings (37–39). Specifically, protofilaments were flexible both within and outside the radial plane, and clusters of laterally connected protofilaments were directly observed as the system was minimizing the mechanical frustration during the relaxation. The nucleotide state affected this delicate balance by modulating both the tangential flexibility of individual protofilaments and the energetics of their lateral interactions. We hence predicted that protofilament clusters might be important structural intermediates that lower the activation barrier for the formation of a straight microtubule lattice. We further hypothesized that kinetic control over cluster formation might be a key determinant of the self-assembly mechanism responsible for dynamic instability of microtubules. However, the computational cost of current atomistic simulations did not allow us to observe reversible association and dissociation of protofilaments into clusters and, therefore, to explore how their lateral interactions would guide the time evolution of a microtubule end at experimentally testable timescales. As a result, there is still no consensus about the true conformational ensemble of the microtubule end and the thermodynamic and kinetic determinants of its capacity to elongate.

But why is it difficult to reach a consensus? Our understanding of the microtubule end dynamics is currently limited by two main challenges. On the experimental side, the transient nature of microtubule ends hinders real-time, high-resolution measurements. Whereas single-particle cryoelectron microscopy offers static snapshots of microtubule segments away from the end with near-atomic resolution, (12, 44–48) variable structures of protofilaments at microtubule ends cannot be resolved to the same extent due to the heterogeneity of their shapes. In turn, cryoET can provide information about the 3D structure of microtubule ends without averaging, (37–39, 49) but with a much lower signal-to-noise ratio, rendering the structural analysis at the level of tubulin–tubulin interactions challenging. Furthermore, fluorescence microscopy can track microtubule ends in real time but lacks spatial resolution to provide structural information (50–53). On the theoretical side, multiscale computational approaches to predict the impact of the bound nucleotide on the dynamics of microtubule ends are not available, while accurate atomistic simulations studying large-scale processes such as fluctuations of the microtubule end are too computationally expensive to cover the relevant timescales. In addition, existing minimalistic models often tend to oversimplify microtubule structure and dynamics, thus limiting the predictive power of these studies. For example, even the most advanced models (38, 40) miss the complex bending-torsional dynamics of protofilaments as well as important correlations caused by intermolecular interactions (41, 42, 54). To obtain a quantitative understanding of structure–dynamics relationships in microtubule assembly, new integrative strategies are therefore required.

In this work, we examine the structure and dynamics of microtubule ends in both nucleotide states at millisecond timescales using coarse-grained (CG) modeling informed and parameterized by atomistic simulations. To this end, an ab initio approach is used to construct an elastic CG model of microtubule end dynamics accounting for both the bending-torsional elasticity of individual protofilaments and the correlations caused by neighbor interactions. We compare results of these simulations with experimental structures of microtubule ends determined using a combination of cryoET and deep-learning-based image denoising, allowing us to increase the precision of segmentation, and thus to determine 3D coordinates of individual tubulin monomers within a flared microtubule end. CG simulations and cryoET reveal that growing microtubule ends feature longer-lived clusters involving a larger number of protofilaments, as compared with the shortening ends. Our modeling also predicts that excess tensile stress in the clusters leads to irreversible protofilament ruptures and tubulin dissociation. Moreover, the rate of these protofilament ruptures is elevated in GTP-state, explaining why growing microtubule ends have shorter and more uniform protofilaments, in agreement with our cryoET measurements. In contrast, GDP-microtubule ends shorten because they get trapped in states with long uneven protofilaments or pairs thereof, thus increasing the free energy barrier that needs to be overcome to form a straight microtubule lattice.

## Results and Discussion

### Protofilament Clusters Are Present at Microtubule Ends in Both Polymerization States.

**Fig. 1.**
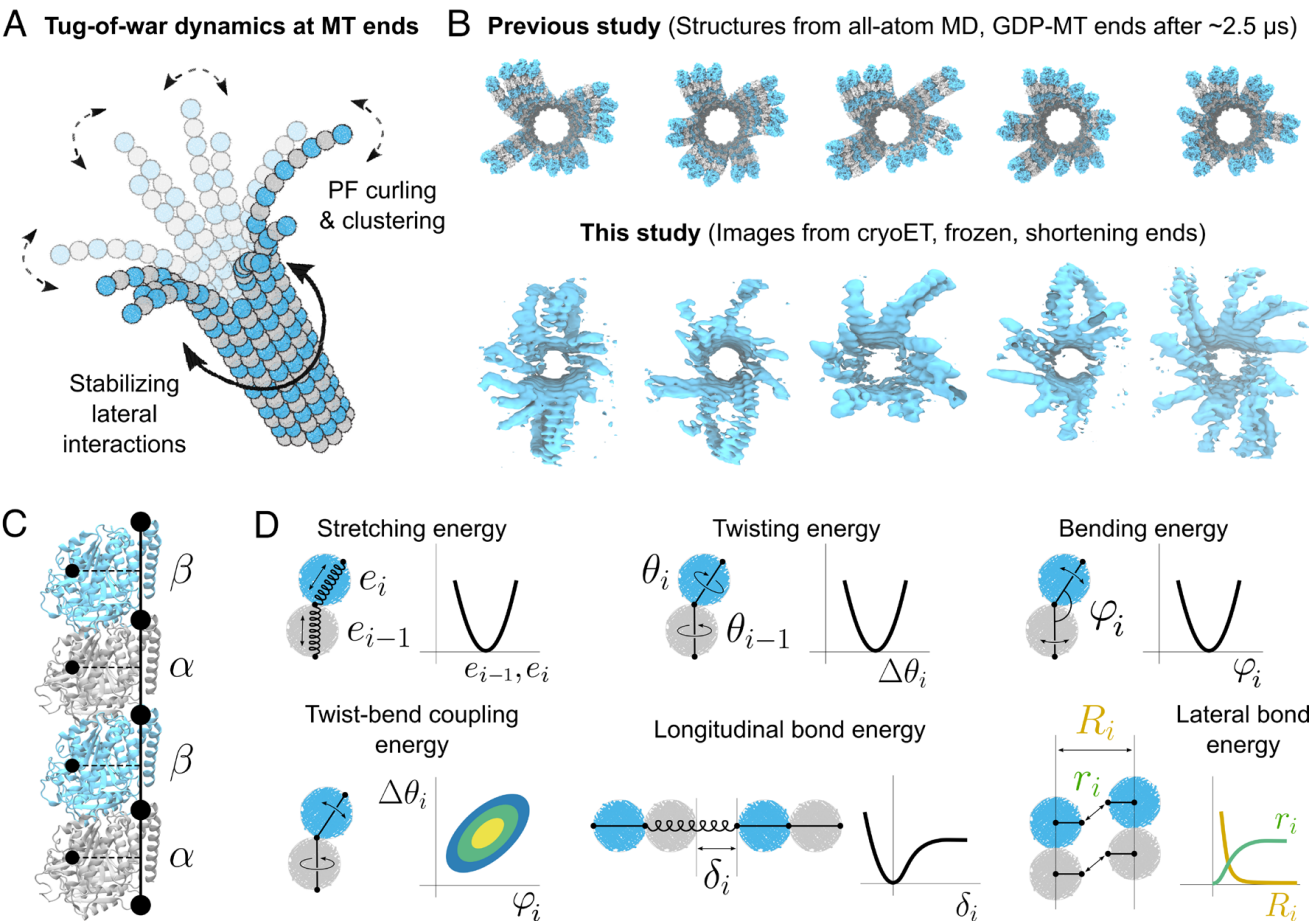
(*A*) Cartoon illustration of the tug-of-war principle: the curved shape of protofilaments at the microtubule end is geometrically incompatible with the straight lattice, resulting in intermediate clusters of partially straightened protofilaments. (*B*) Comparison of the simulated GDP-microtubule ends from our previous study (42) (*Top*) with exemplary 3D rendered volumes of shortening microtubule plus-ends obtained in this study (*Bottom*). (*C*) CG representation of a protofilament (black circles with solid and dashed lines) mapped onto its atomistic structure. α- and β-tubulin are shown as gray and cyan ribbons, respectively. (*D*) Minimal elastic coarse-grained model of a microtubule end. Each protofilament is modeled as a set of nodes connected by stretchable and twistable springs. Coupling between bending and twisting of neighboring strings is introduced to better reproduce the atomistic dynamics. All deformations are described by harmonic potentials except those between individual tubulins.

Our previous all-atom molecular dynamics (MD) study (42) showed that the nonequilibrium relaxation of the microtubule end structure occurs at microsecond time scales and is driven by a “tug-of-war” between the bending-torsional elasticity of protofilaments and lateral interactions between them (Fig. 1*A*). However, neither was it possible to converge these computationally expensive simulations to a steady state in which the processes of protofilament clustering and separation would be in equilibrium, nor were experimental structural data supporting this hypothesis available. Here, we ask whether protofilament clusters generally exist at the ends of growing and shrinking microtubules and, if so, whether a model can be derived to accurately describe and understand this phenomenon from fundamental principles.

To resolve the structures of microtubule ends in growing and shortening states, we performed cryoET on samples containing dynamic microtubules polymerized from purified porcine brain tubulin (*Materials and Methods*). Cryo-CARE denoising allowed us to reduce high-frequency noise sufficiently enough to resolve individual flaring protofilaments at microtubule plus-ends in 3D (55). Since the microtubules were polymerized from GMPCPP-stabilized seeds, the majority of them had 14 protofilaments, which enabled an unambiguous comparison of the reconstructed tomograms with our all-atom MD simulations of 14-protofilament microtubule ends. Fig. 1*B* shows the end-on view of protofilament flares of the GDP-microtubule plus-ends simulated for ~2.5 µs from our previous study and some exemplary 3D tomograms of the shortening microtubule plus-ends obtained in this study (see *SI Appendix*, Fig. S1*A* for the comparison of growing microtubule ends).

Even without further analysis of the tomograms, which will be presented in detail below, we could clearly observe protofilament clusters as the resolution was sufficient to observe individual tubulin molecules. Contrary to previous reports, (37, 38) the protofilaments in our samples deviated from their radial planes to form clusters with their neighbors–an observation which we attribute to the improved image processing. Our previous MD simulations (Fig. 1 *B*, *Top* row) already established that a soft tangential mode of protofilament motion was responsible for the out-of-plane deviations [“tangential swing”; see Movie S3 in here (42)]. Furthermore, approximately 84% of the reconstructed microtubule ends showed a global left-handed twist pattern, i.e. the protofilaments twist-bent counterclockwise in the direction of microtubule growth. This pattern–also clearly observed in the simulated microtubule ends–likely resulted from the torsional component in the protofilaments’ main bending mode [see Movie S3 in here (42)], which caused asymmetric exploration of the conformational space at the microtubule end. Together, these observations lead us to conclude that protofilament clusters are not an artifact of cryoET reconstructions or simulations, but rather structural intermediates characteristic of both microtubule polymerization states.

### A Coarse-Grained Model Allows Access to Submillisecond Dynamics of the Microtubule End.

We constructed an elastic CG model to quantify the dynamics and energetics of protofilament clustering at the microtubule end. To account for the radial and tangential elasticity of protofilaments, each tubulin dimer was represented by three CG beads connected by stretchable and rotatable springs (Fig. 1*C*; see *Materials and Methods*, *SI Appendix*, Fig. S1*B* and Table S1 for a detailed description of the model geometry). Thus, the black dots in Fig. 1*C* represent sites at which model parameters are relevant, while the minimal simulated entity in our model is a tubulin dimer (three consecutive beads). This type of bead assignment reflects the fact that protofilaments bend and twist at “hinge” regions located at the intra- or interdimer interfaces between α- and β-subunits (42, 54, 56). Therefore, unlike in many previous models, a CG bead in our model does not coincide with a tubulin monomer but instead is shared by two neighboring monomers except when it is a terminal one. Unless stated otherwise, all elementary deformations in the model were designed to be harmonic, and the corresponding mechanical parameters for every triplet of CG beads were set to depend only on the nucleotide state and on whether it described an intra- or interdimer interface. To describe protofilament–protofilament interactions and to allow for tubulin dissociation, we also introduced breakable lateral and longitudinal bonds. Fig. 1*D* schematically summarizes the elementary strains, and the graphs beside them show the potential functions under consideration. The model parameters were derived from our previous (42) and newly produced all-atom MD simulations (see *SI Appendix*, Fig. S1 *C*–*E* and Table S2 for a detailed description of the parameterization procedure). We note that, unlike in classical kinetic models, (30, 57, 58) which describe microtubule polymerization using empirically derived kinetic rates and binding affinities, our model was developed with a different goal in mind: capturing mechanical deformations and dynamics at the microtubule end at scales that traditional biochemical models or atomistic MD cannot access. Because of this, our model parameters do not straightforwardly correspond to any traditional kinetic descriptors such as binding affinities or kinetic rates.

### Nucleotide State Modulates Tug-of-War Dynamics by Controlling Protofilament Cluster Size, Number, and Stability.

The model introduced above allowed us to predict the stochastic time evolution of the microtubule end at submillisecond timescales currently inaccessible to both high-resolution microscopy and all-atom MD simulations. It further enabled a direct comparison to the structures obtained via cryoET (which are presented in detail below). In our previous MD study, (42) we predicted that the size and stability of intermediate protofilament clusters should determine the probability of growth or shortening. Consequently, larger and more stable clusters should increase the growth probability without significantly changing the overall flared shape of the microtubule end. To test this prediction, we carried out Brownian Dynamics simulations of our model to obtain conformational ensembles of microtubule ends with 6 dimers per protofilament as a function of the nucleotide state and the lateral interaction energy. For each pair of these parameters, approximately 30 × 200 µs of CG trajectories were generated (see *Materials and Methods* and *SI Appendix* *f*or details on simulations and analysis and Movies S1 and S2 for exemplary trajectories of the GTP- and GDP-microtubule end dynamics).

**Fig. 2.**
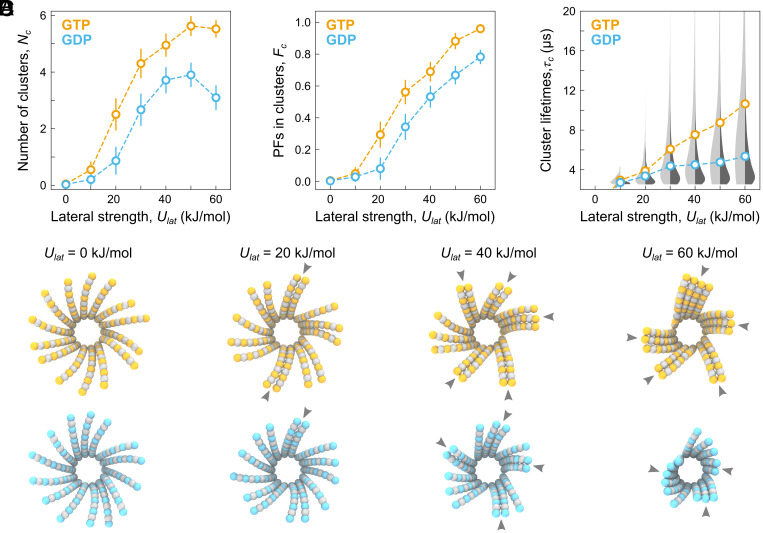
(*A*) The average number of clusters, *N_c_*, (*B*) the average fraction of protofilaments in clusters, *F_c_*, and (*C*) the average cluster lifetime, *τ_c_*, plotted as a function of nucleotide state (orange for GTP and cyan for GDP) and lateral interaction energy, *U_lat_*. For *N_c_* and *F_c_*, the error bars indicate SD calculated over all time frames (*n* = 8,000) and all simulation replicas (*m* = 30). For *τ_c_*, no error bars are provided; instead, full statistical distributions (light gray for GTP, dark gray for GDP) are shown overlaid with the average lifetimes. The length of protofilaments was fixed and equal to 6 dimers in all simulations. (*D*) Top view of representative microtubule end structures in both nucleotide states for selected lateral interaction energy values. Gray arrowheads indicate protofilament clusters.

We first quantified how many clusters were formed and what fraction of protofilaments participated in clustering for both nucleotide states. As expected, in the absence of lateral interactions (*U_lat_* = 0 kJ/mol, where *U_lat_* is the attractive part of the lateral interaction potential in Fig. 1*D*), the protofilaments did not form clusters. With increasing *U_lat_*, an increasing number of protofilament clusters emerged (Fig. 2*A*) that also gradually grew in size (Fig. 2*B*). For very strong lateral interactions *U_lat_* > 50 kJ/mol, the clustering was limited by the number of available protofilaments in the microtubule (14 in our simulations), reflected in a slight drop in the average number of clusters.

We next calculated the distribution of protofilament cluster lifetimes from the moment they formed to their complete or partial dissociation (Fig. 2*C*). We observed larger lifetimes and, consequently, broader lifetime distributions for increasing *U_lat_*. Interestingly, the size, the number, and the average lifetime of clusters were consistently smaller for the GDP-microtubule ends compared to the GTP-microtubule ends, irrespective of the lateral interaction energy (Fig. 2 *A*–*C*). We speculate that soft and flexible GTP-protofilaments explore a larger tangential spatial range, hence increasing their probability to encounter a neighbor protofilament and to form/join a cluster. This behavior is directly linked to the nucleotide state, which modifies the dynamics of the intra- and interdimer interfaces (42, 54). Fig. 2*D* visualizes the described quantitative trends by showing representative snapshots of microtubule ends in both nucleotide states.

We also observed that larger protofilament clusters tend to adopt straighter conformations, supporting our hypothesis that protofilament clusters are polymerization intermediates. First, the free energy required to straighten a protofilament cluster decreases rapidly with the cluster size (*SI Appendix*, Fig. S2*A*). Second, the average curvature gradient between a protofilament’s tip and its attachment point at the microtubule shaft increases with increasing *U_lat_* (*SI Appendix*, Fig. S2*B*), which qualitatively reproduces the curvature analyses in the original studies by McIntosh, Gudimchuk et al (37, 38). This effect is also expected within the tug-of-war concept: The microtubule end gains additional energy by forming lateral bonds between neighboring protofilaments; this energy is then spent on “forcing” the protofilament clusters into straighter conformations away from their equilibrium shape.

Altogether, these results demonstrate that a wide spectrum of statistical distributions of protofilament cluster numbers, sizes, and lifetimes can be achieved by testing physically relevant ranges of the spring-like parameters of protofilaments and their lateral interaction energies. Moreover, the behavior of the simulated microtubule end within these parameter ranges does not result in large-scale conformational changes in the flared structure of microtubule ends, which aligns well with previous (37–39) and our own cryoET measurements (Fig. 1*B* and *SI Appendix*, Fig. S1*A*). It is hence plausible that GTP-microtubule ends that form more, larger, and longer-lived protofilament clusters are also more polymerization-prone because their statistical ensemble is more similar to a fully straight lattice.

### Nucleotide State Affects Protofilament Length via Asymmetric Strain in Clusters and Protofilament Ruptures.

To avoid studying finite size effects, we asked whether and how the dynamics and the distribution of clusters would change with the length of protofilaments, *L_PF_*. To this end, we repeated the above simulations for *L_PF_* between 3 and 9 dimers and calculated 2D parametric diagrams for the average number of clusters (*SI Appendix*, Fig. S3 *A* and *B*) and the average fraction of protofilaments in clusters (*SI Appendix*, Fig. S3 *C* and *D*). These simulations showed that, for small to moderate lateral strengths (*U_lat_* ≲ 40 kJ/mol), the propensity to form clusters generally decreased with increasing *L_PF_*. This implies that for small *L_PF_*, the free energy spent on straightening the protofilaments, and the free energy gained from forming lateral bonds between the protofilaments approximately compensate each other, enabling more frequent cluster formation. At the same time, the straightening energy increases faster with *L_PF_* than the lateral energy decreases, shifting the balance toward more splayed end conformations for large *L_PF_*. We believe that there are two reasons for the faster increase of the curved-to-straight free energy barrier: i) a higher enthalpic contribution due to twist accumulation in longer protofilaments, and ii) a higher entropic contribution due to a larger phase space available to longer protofilaments.

**Fig. 3.**
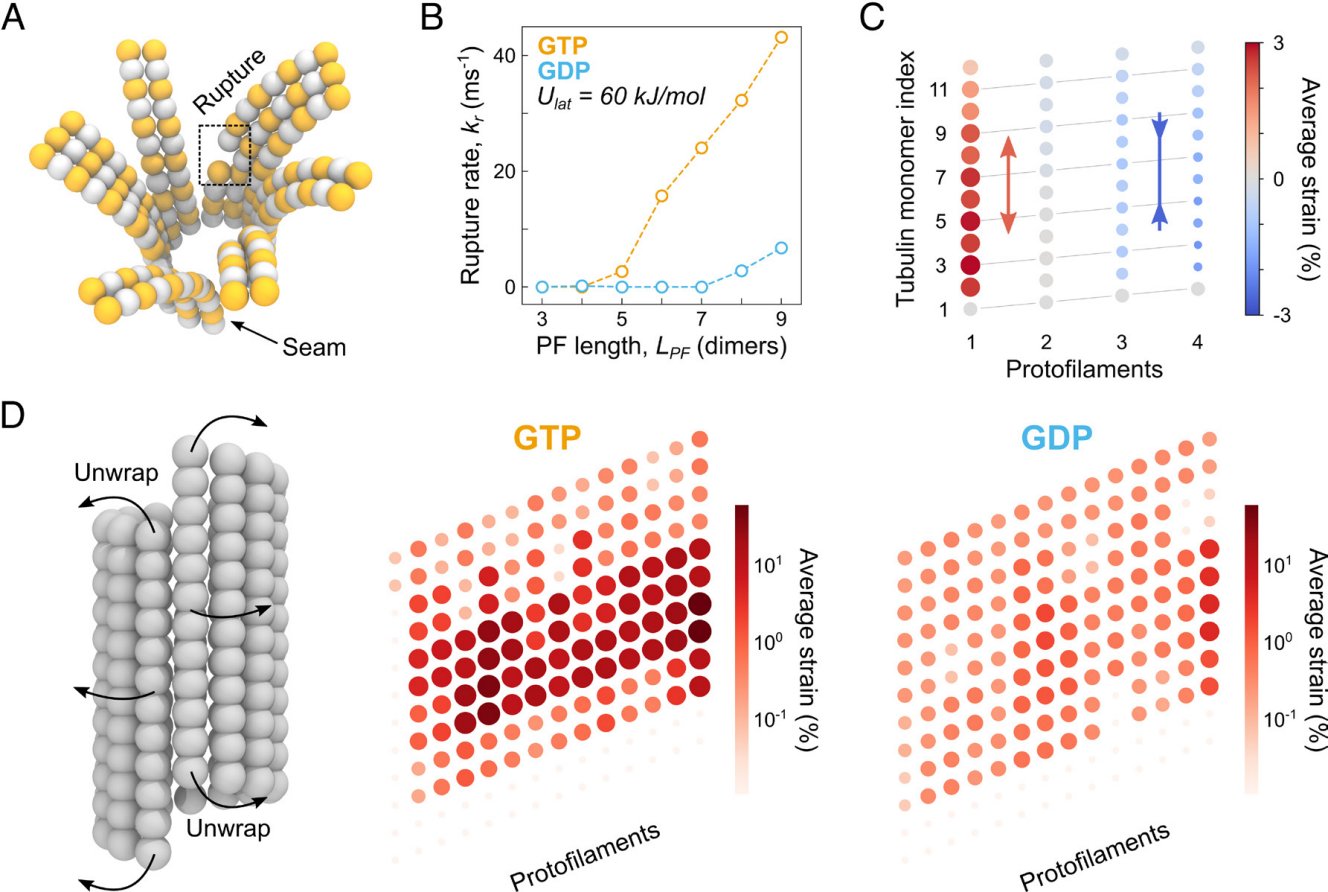
(*A*) Snapshot from one of our CG simulations demonstrating a rupture event (dashed square). (*B*) The rate of rupture events *k_r_* plotted as a function of protofilament length *L_PF_* and nucleotide state (orange for GTP and cyan for GDP). The lateral interaction strength was fixed at *U_lat_* = 60 kJ/mol (see *SI Appendix*, Fig. S4 *A* and *B* for the full 2D parametric diagrams). (*C*) The average relative strain along GDP-protofilaments in a cluster of size 4 and *L_PF_* = 6 dimers relative to that in the initial straight configuration. Each circle corresponds to a tubulin monomer while its color and size denote the magnitude and the sign of strain, respectively. The lateral and longitudinal bonds were replaced with harmonic potentials to prevent dissociation. (*D*) The average relative strain along protofilaments in a full GTP- and GDP-microtubule lattice of *L_PF_* = 6 dimers relative to that in the initial straight configuration, when unwrapped onto a 2D lattice representation. The lateral interaction strength was fixed at *U_lat_* = 60 kJ/mol. Each circle corresponds to a tubulin monomer while the color and size denote the magnitude of strain. Log-scale was chosen to emphasize the difference in mechanical frustration between GTP- and GDP-microtubule ends.

For high lateral strengths (*U_lat_* ≳ 40 kJ/mol), the propensity to form clusters remained constant or even increased with increasing *L_PF_*; however, the simulated microtubule ends were increasingly distorted and unstable. When inspecting simulation trajectories, we observed events in which protofilaments at the edges of clusters would spontaneously rupture and dissociate. Such rupture events were rarely observed during the characteristic lifetime of protofilament clusters (Fig. 2*C*) but still occurred sufficiently often within the 30 × 200 µs timescale of our simulations. Interestingly, these events mainly occurred on the left side of a cluster when viewed from within the lumen (see the example in Fig. 3*A*). This unexpected observation prompted us to study the statistics of rupture events in more detail.

First, we quantified how frequently ruptures occurred in the GTP- and GDP-microtubule trajectories depending on *L_PF_*. While only very few ruptures were seen for both nucleotide states when *U_lat_* ≲ 40 kJ/mol, the rupture rate in GTP-microtubule ends steeply increased at *U_lat_* ≳ 40 kJ/mol and *L_PF_* ≳ 5 dimers (Fig. 3*B*; see *SI Appendix*, Fig. S4 *A* and *B* for the full 2D parametric diagrams). Considered together with Fig. 2 *A*–*C* and *SI Appendix*, Fig. S3, these data suggest that cluster formation might be linked to protofilament rupture and subsequent dissociation of tubulin dimers or oligomers via a negative feedback mechanism.

To test this idea, and in particular the reciprocal causal relationship, we constructed GTP-clusters of size between 1 and 4 protofilaments and performed independent simulations of these clusters for different *L_PF_*. To keep the clusters intact over the course of the simulation, we made all lateral and longitudinal bonds harmonic. Fig. 3*C* shows the average distribution of longitudinal mechanical strain in a cluster of size 4 protofilaments and *L_PF_* = 6 dimers. This distribution was strongly asymmetric, with the highest stretching strain localized on the left side (where most ruptures had occurred in our simulations) and the highest compression strain localized on the right side of the cluster when viewed from within the lumen. The asymmetry became more pronounced with increasing either *L_PF_* or the cluster size (see *SI Appendix*, Fig. S5 for the full 2D parametric diagrams). We speculate that this asymmetric strain in clusters is caused by the special “hinge” structure connecting dimers in each protofilament, which enables (i) compression/extension (59) and (ii) torsional-bending coupling (42, 54) along the protofilament axis. While the torsional-bending coupling makes each protofilament deviate from its radial plane, the longitudinal strains propagate across the protofilaments through lateral contacts. Thus, these simulations of “indestructible” clusters demonstrate that excess stretching strain correlates with the location of protofilament rupture, regardless of the cluster size and *L_PF_*.

Finally, we quantified how the longitudinal mechanical strain causing protofilament rupture was distributed across the complete microtubule end and how it depended on the nucleotide state. To this end, we reanalyzed the simulation datasets shown in Fig. 2 as follows: Each trajectory was truncated just before the first rupture occurred, and the average strain per dimer was calculated over all the independent and truncated trajectories. Unexpectedly, unlike in the single cluster case (Fig. 3*C*), the average strain in the entire microtubule was predominantly positive, i.e. the protofilaments were, on average, overstretched relative to their initial straight configurations. More specifically, the maximum average strain measured was +58.3% and +4.0% for the GTP- and GDP-microtubule ends, respectively, while the minimum average strain was less than −0.04% in both cases. To visualize the difference between the strain distributions in the GTP- and GDP-microtubule ends more clearly, we neglected the small fraction of negative strains and used a log-scale (Fig. 3*D*). The GTP-microtubule lattice was, on average, much more mechanically frustrated–despite the known increased radial and tangential softness of its protofilaments (42). Moreover, the localization of excess strain within the GTP-microtubule end coincided well with the most frequent location of ruptures, namely near the lattice shaft and distant from the protofilament tips. We believe that this is because (i) protofilaments are softer to stretching deformations than compressing ones, (59, 60) and (ii) unlike in the simulations of isolated clusters shown in Fig. 3*C*, the lateral and longitudinal bonds are breakable in the whole microtubule end simulations. This observation further indicates that, despite the asymmetric deformation behavior localized in isolated indestructible clusters, the short-lived nature of clusters in a more realistic, full microtubule simulation (Fig. 2), combined with protofilament exchange among them, leads to an almost uniform probability distribution of longitudinal bond breakages across the protofilaments, leading to a more even distribution in *L_PF_*.

Taken together, these data demonstrate an interesting phenomenon: While GTP-microtubule ends form larger and longer-lived protofilament clusters more frequently (Fig. 2 and *SI Appendix*, Fig. S3), the resulting excess mechanical frustration in the clusters leads to more frequent protofilament ruptures (Fig. 3 and *SI Appendix*, Figs. S4 and S5), thus affecting the shape of the microtubule ends. It is conceivable that the ability to form and maintain sufficiently large protofilament clusters correlates with a reduction of *L_PF_* and vice versa. The fact that this reciprocal relationship is strongly nucleotide-dependent (*SI Appendix*, Figs. S3 and S4) sets constraints on the potential mechanism of microtubule assembly. In particular, because GTP-microtubule ends favor configurations with large and long-lived clusters, which decreases the average *L_PF_*, newly incoming dimers are more likely to form a sufficient number of lateral bonds to get accommodated into the lattice. Conversely, a gradual loss of the GTP cap due to hydrolysis shifts the conformational preference toward smaller and less stable clusters, which reduces the mechanical frustration but increases the average *L_PF_*, thereby raising the free energy barrier to convert individual protofilaments into a straight microtubule lattice.

### Electron Tomography Confirms Predicted Structural Differences at the Ends of Growing and Shortening Microtubules.

The self-assembly mechanism proposed above enables several predictions that can be tested experimentally. First, the ends of growing microtubules should have shorter protofilaments. Second, they should have more protofilament clusters. Third, the difference in *L_PF_* as well as the difference in the frequency and the size of clusters between growing and shortening microtubule ends should be significant yet subtle, because more pronounced differences would render dynamic instability energetically unfeasible.

**Fig. 4.**
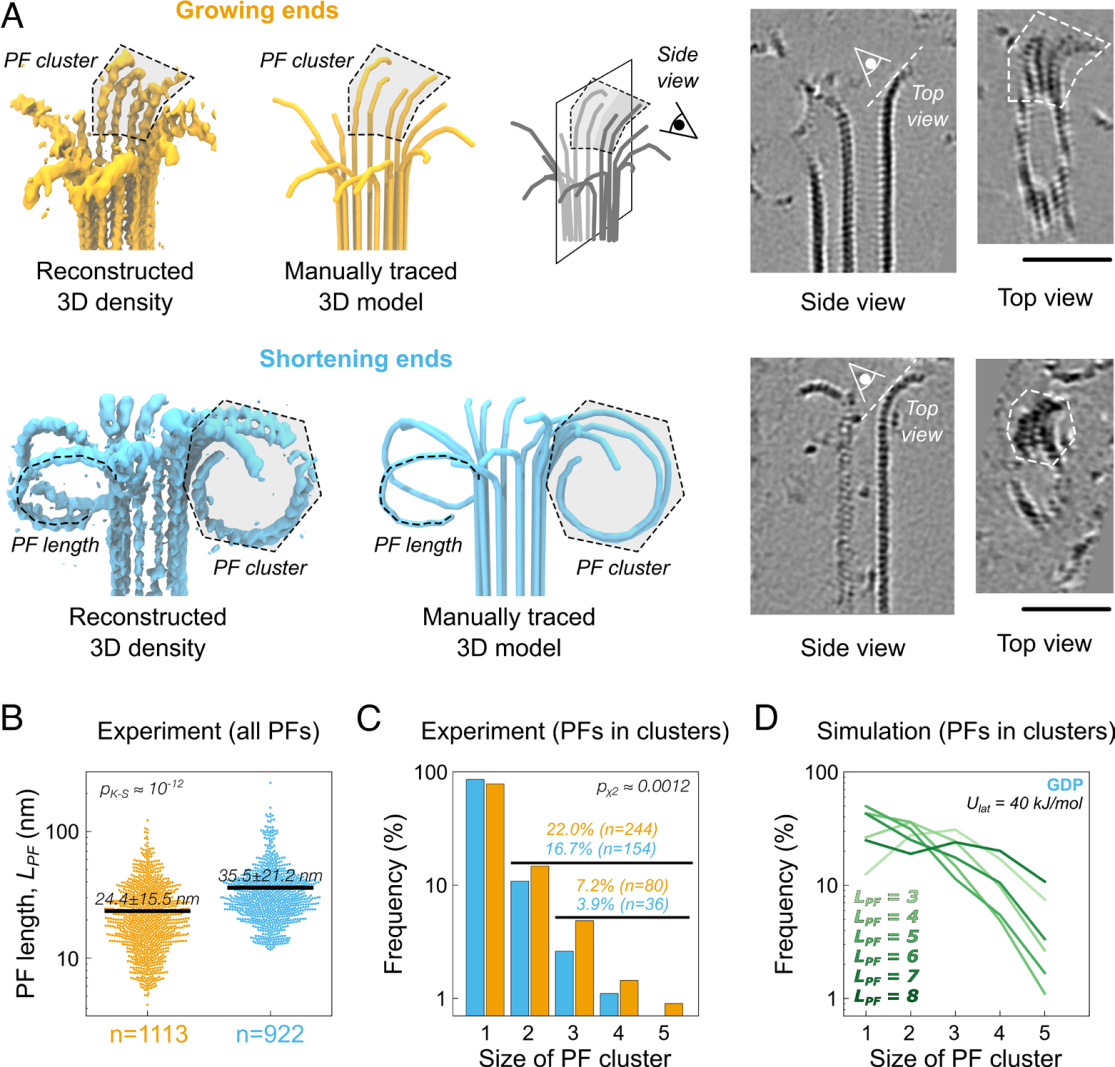
(*A*) Segmented and 3D rendered volumes and manually traced 3D models showing growing (orange, *Left*) and shortening (cyan, *Right*) microtubule ends. A typical protofilament cluster is marked with a light gray dashed area. Also shown are tomographic slices with a cluster in cross-section (side view), and parallel to the initial segment of a cluster flaring out of the microtubule cylinder (*Top* view). Also note another partially formed microtubule lattice in the tomographic image of the growing microtubule end. These formations are rarely observed in our samples and are not included in our analyses. (Scale bar, 50 nm.) (*B*) Distribution of protofilament lengths for cryoET samples imaged in the presence of soluble tubulin under growing (orange) and shortening (cyan) conditions. Shown are raw data points (dots) corresponding to individual protofilaments and mean values (black lines). Log-scale was chosen to visualize the two distinct distributions on a single scale. The value in the upper left corner reports the results of a Kolmogorov–Smirnov test and its *P*-value. (*C*) Distribution of protofilament cluster sizes including single protofilaments from the experimental datasets shown to the left. The value in the upper right corner reports the results of Pearson’s $\chi^{2}$ test and its *P*-value. The values *n* report the numbers of individual protofilaments in a category (all clusters and large clusters of 3 or more protofilaments). (*D*) Same as in (*C*) but calculated from the simulated ensembles of GDP-microtubule ends shown in Fig. 2. Shown are the cluster size statistics for multiple *L_PF_* and *U_lat_* = 40 kJ/mol.

To test these three predictions, we performed cryoET using dynamic microtubules reconstituted from purified tubulin and GMPCPP-stabilized seeds attached to electron microscopy grids. To image growing microtubules, we froze the grids after several minutes of incubation with high tubulin concentration. To image shortening microtubules, we diluted tubulin to below 2 μM and froze the grids after 30 to 45 s. We then performed cryoET and denoised the tomograms using the Cryo-CARE approach (55). After determining the polarity of microtubules, we manually segmented plus ends and traced their protofilaments (Fig. 4*A* and *SI Appendix*, Fig. S6; see *Materials and Methods*). From these 3D traced models, we first obtained the samples of *L_PF_* measured from the first segment bending away from the microtubule cylinder to the protofilament’s tip (n = 1,113 and n = 922 for growing and shortening end, respectively). Analysis of these samples revealed a wide distribution that was shifted toward longer protofilaments for shortening microtubule ends (Fig. 4*B*), consistent with previous reports (37, 38). A two-sample Kolmogorov–Smirnov test additionally showed that the two samples do not belong to the same unknown distribution (statistic $D_{K-S}=0.17$ and *P*-value $P_{K-S}\approx 10^{-12}$).

Further, we excluded all protofilaments that had no proximal neighbors based on their normalized mutual overlap (see *Materials and Methods* and *SI Appendix*, Fig. S7) and interpreted the remaining fraction as protofilament clusters. We sorted all protofilaments according to the size of the clusters they belonged to and constructed a contingency table summarizing the counts for all cluster sizes in both polymerization states. We only considered neighboring protofilaments as clustered if they interacted along their entire lengths. We observed 22.0% (n = 244) and 16.7% (n = 154) of all protofilaments in clusters at growing and shortening microtubule ends, respectively (Fig. 4*C*). Additionally, 7.2% (n = 80) of protofilaments at growing ends formed large clusters with three or more protofilaments, whereas only 3.9% (n = 36) of large clusters were observed at shortening ends. Pearson’s $\chi^{2}$ test confirmed that the difference in the observed counts of protofilaments belonging to clusters of a particular size at growing or shortening microtubule ends is statistically significant (statistic $\chi^{2}=18.1$ and *P*-value P≈0.0012).

We also computed the cluster size statistics from our simulations. Fig. 4*D* shows the distribution of cluster sizes for the GDP-microtubule ends simulated at *U_lat_* = 40 kJ/mol and for different *L_PF_*, demonstrating that it is, in principle, possible to select such a pair of *U_lat_* and *L_PF_* for our model to approximately reproduce the experimental values (Fig. 4*C*, cyan). Despite this favorable agreement, we must note that it is not fully quantitative because, while in our simulations all protofilaments have the same length, in experiments, the protofilament length distribution at the microtubule end is very ragged (Fig. 4*B*). Nevertheless, it is remarkable that the experimentally observed cluster sizes lie well within the range covered by our CG simulations. Finally, whereas the distributions of both *L_PF_* and cluster sizes differ only subtly (Fig. 4 *B* and *C*), we have now been able to detect this difference and show its statistical significance, thanks to the improved resolution provided by Cryo-CARE. Taken together, our cryoET results are in a remarkable agreement with the predictions delivered by the CG simulations.

## Conclusions

**Fig. 5.**
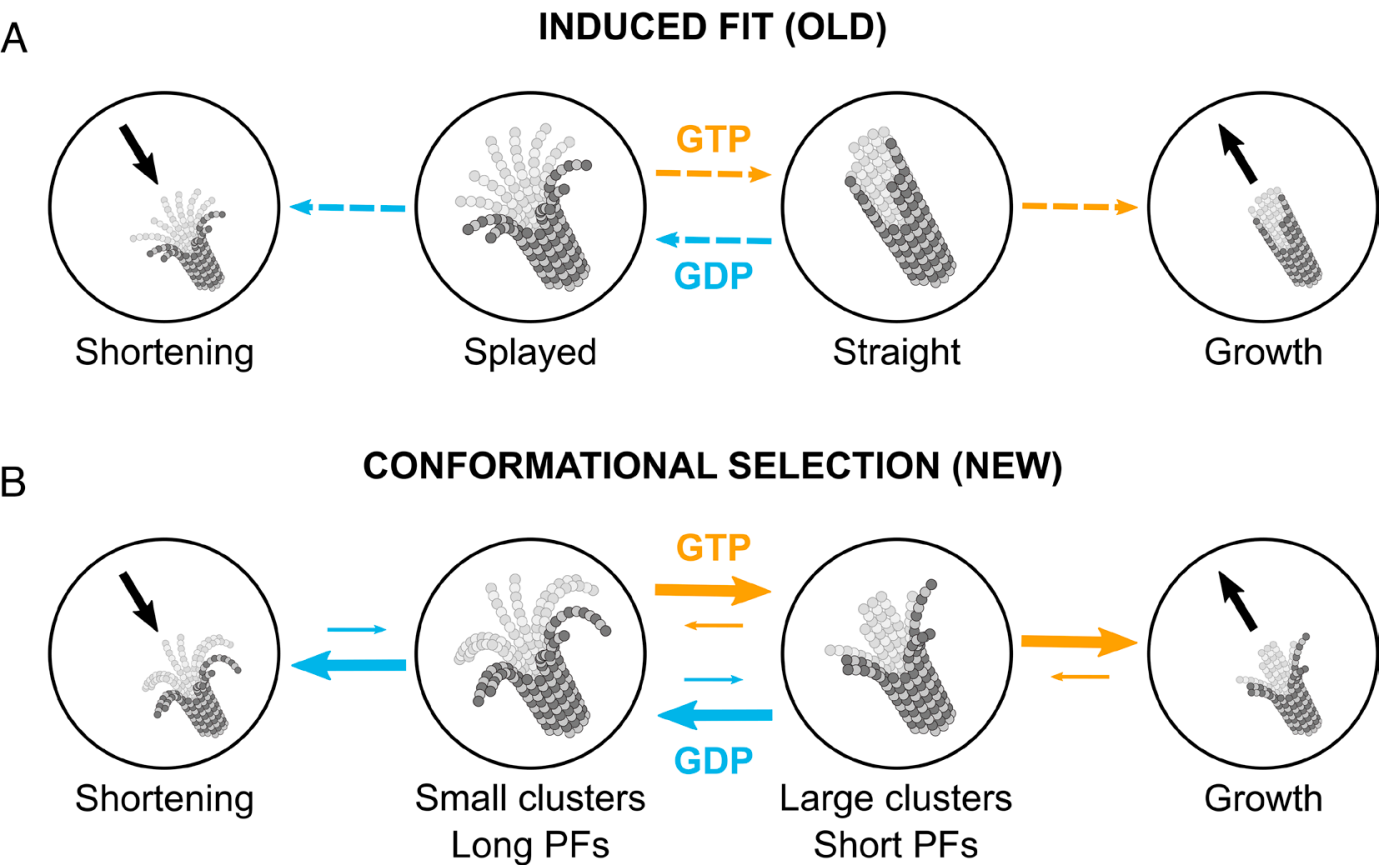
Schematic illustration of the induced fit (*A*) and the conformational selection (*B*) mechanisms. Note that the solid arrows in (*B*) denote kinetic rates according to our model, where thicker and longer arrows correspond to higher kinetic rates. In contrast, the dashed arrows in (*A*) simply show the direction of preferred transitions based on the nucleotide state.

Based on our findings presented here, we propose a mechanism of microtubule assembly, which we term *conformational selection*. It synergizes the previous cryoET experiments by McIntosh, Gudimchuk, and others (2, 37–39) as well as the large-scale atomistic MD simulations of microtubule ends (41–43). Fig. 5 schematically illustrates its key differences to the previously proposed mechanism, which we term *induced fit* for consistency.

The induced fit mechanism (Fig. 5*A*) postulates that the shape of protofilaments at microtubule ends is directly controlled by the nucleotide state (16–19). A growing microtubule end is inherently straight and blunt so that the assembly mainly occurs through cooperative addition of straight GTP–tubulin dimers. Upon GTP hydrolysis, the microtubule end begins to coil inside-out and shorten, producing curled and splayed GDP-protofilaments and curved GDP–tubulins in solution.

In contrast, we propose that irrespective of the nucleotide state, all microtubule ends are splayed and can form protofilament clusters (Fig. 1*B* and *SI Appendix*, Fig. S1*A*) driven by the tug-of-war between protofilament elasticity and lateral interactions (41, 42) (Fig. 1*A*). The nucleotide state modulates the mechanical flexibility of each protofilament, resulting in larger (GTP) or smaller (GDP) clusters (Fig. 2 and *SI Appendix*, Fig. S3). Clusters of long and soft GTP-protofilaments are subject to excess mechanical strain and protofilament ruptures (Fig. 3 and *SI Appendix*, Figs. S4 and S5), leading to GTP-protofilaments being, on average, shorter but forming larger clusters than GDP-protofilaments (Fig. 4). These fast, microsecond tug-of-war dynamics, combined with tension-induced, nucleotide-dependent protofilament ruptures at submillisecond timescales, as well as tubulin binding/unbinding at millisecond timescales (20 to 40 ms (51, 61) at 10 µM tubulin) drive the conformational selection for polymerization-prone configurations showing higher similarity to a straight microtubule end (Fig. 5 *B*, *Middle*). GTP hydrolysis reduces the probability of growth by decreasing the average cluster size, resulting in long “ram’s horn”-like GDP-protofilaments and pairs thereof, thereby triggering microtubule catastrophe. Besides providing striking agreement with our cryoET data (Fig. 4), this model emphasizes and explains the stochastic nature of microtubule self-assembly and catastrophe driven by GTP hydrolysis.

Cooperative assembly of microtubules has been traditionally explained by “cozy corner” models in which the incorporation of straight tubulins at growing blunt microtubule ends is facilitated by simultaneously forming lateral and longitudinal bonds (62). This view has been challenged by the original studies by McIntosh, Gudimchuk, and others (37–39). In this context, Erickson has recently introduced the idea of a “conformational switch” to explain how the cooperativity can still occur within this framework (63). It postulates that tubulin is able to transition from a low- to a high-affinity state upon binding to the tip of a single protofilament, forming a longitudinal bond. This transition remains a hypothesis and, to the best of our knowledge, has not been confirmed experimentally. Instead, our model presents a unifying perspective by proposing that microtubule assembly occurs via clusters of protofilaments, which aligns with the flared microtubule end structure in both polymerization states and could still provide cozy corner-type binding sites due to different protofilament lengths in clusters, while preserving the bent conformation of tubulin. Furthermore, our conformational selection mechanism resonates with the idea of cooperative tubulin straightening at the microtubule end and hydrolysis-induced strain propagation in the microtubule lattice, (64) suggesting that the straightening (or compaction/extension) of one dimer both requires and facilitates the straightening (or compaction/extension) of its neighbors. Using advanced modeling combined with state-of-the-art cryoET, our study now demonstrates exactly how this allosteric effect is achieved.

This conformational selection mechanism may help explain microtubule behavior in various physiological and pathological contexts or prompt reassessment studies of fundamental aspects of microtubule dynamics that are now taken for granted. Important examples include the regulation of microtubule dynamics by force and microtubule-associated proteins. For example, tensile forces stabilize the kinetochore–microtubule attachment in mitosis, stall microtubule disassembly, and induce microtubule rescue (65, 66). The kinetochore could bundle and straighten neighboring GDP-protofilaments of the disassembling microtubule end into a more GTP-like state (increased clustering), which could provide a possible explanation for the kinetochore’s ability to remain attached to microtubule ends under force (67–69). Further, the conformational selection mechanism could offer a leverage for multivalent microtubule-binding oligomers of the human Ndc80 or budding yeast Dam1 complexes to modulate the polymerization dynamics by controlling protofilament clusters, without either the microtubule end or the kinetochore having to undergo large conformational changes.

The conformational selection mechanism could also explain why human end-binding proteins EB1/3 and their fission yeast homologues Mal3 accelerate microtubule growth (70, 71). Since both bind microtubule lattices in between neighboring protofilaments, (45, 72) we speculate that they either directly promote the formation of new protofilament clusters or stabilize existing or potentially emerging protofilament clusters against dissociation. The latter scenario appears more plausible as EB1 and its homologues have a higher affinity for a hydrolysis intermediate of GTP–tubulin, with the maximum occupancy site located slightly behind the growing end, (70) a location to which they recruit other proteins in massive comet-like assemblies (73, 74). Within the conformational selection model, binding of EB1 would shift the dynamic tug-of-war equilibrium toward a straighter microtubule lattice and, thus, accelerate conformational maturation of the growing end.

Finally, binding of taxol has been shown to stabilize microtubules below or near stoichiometric equivalence with tubulin dimers both in vitro and in vivo; (75) however, the stabilization mechanism is still debated. More recently, taxol binding has been shown to invert the conformational change that normally occurs in response to GTP hydrolysis, producing expanded and more heterogeneous microtubule lattices (44, 76). We and others have also confirmed in a series of atomistic MD studies that expanded tubulin conformations result in softer protofilaments and microtubule lattices (42, 54, 59). Assuming that these softening effects are hallmarks of a GTP-like state of tubulin, we speculate that taxol binding increases protofilament radial and tangential flexibility, shifting the equilibrium toward larger clusters of shorter protofilaments. Indeed, a recent cryoET study has reported that growing microtubule ends treated with 10 nM of taxol feature even shorter protofilaments than growing microtubule ends in the control experiment, (38) though protofilament clusters have not been analyzed.

This type of regulation by modulating the stability of self-assembly intermediates (protofilament clusters) might not be unique to taxol. Microtubules have been reported to disassemble faster in the presence of high concentrations of Mg^2+^, and that adding substantial amounts of Mg^2+^ creates longer protofilament curls (4, 77, 78) and increases the work transferred by these curls in optical tweezers assays (7). Thus, it cannot be ruled out that certain mutations not interfering with lateral and longitudinal lattice interfaces or the nucleotide binding pocket exploit the conformational selection mechanism to alter microtubule dynamic instability, for example β:T238A causing faster growth in hyperstable yeast and human microtubule phenotypes (79, 80) or β:D417H/β:R262H linked to ocular motility disorder in humans and also causing faster growth (81).

From the simulation perspective, our CG model (Fig. 1 *C* and *D*; see also *SI Appendix*, Fig. S1, *Materials and Methods*) accounts for a number of structural and dynamical properties that were previously obtained through accurate atomistic MD simulations of tubulin oligomers and complete microtubule ends (42). These simulations set strict physical constraints on the type of degrees of freedom and the nature and strength of tubulin–tubulin interactions. For example, previous CG models did not allow for the possibility of protofilaments deviating from their radial planes and did not consider the bending-torsional coupling within each protofilament, and therefore did not predict intermediate protofilament cluster states. Here, we show that a rigorous, discrete elastic model optimized against high-resolution atomistic simulations can overcome this limitation and reach excellent agreement with experiment. Nevertheless, our CG model still contains a number of approximations. First, our CG mapping (Fig. 1*C* and *SI Appendix*, Fig. S1*B*) might miss important degrees of freedom that have not yet been resolved by electron microscopy or have been observed in all-atom MD but not recognized as functionally relevant. Second, in our CG model, all elementary protofilament deformations are harmonic, and only local correlations are considered, neglecting nonlinear mechanical effects or long-range interaction components. Third, although our CG model has been parameterized using extensive atomistic MD trajectories (~400 µs of cumulative sampling), these do not account for potentially relevant conformational changes occurring at longer timescales. Fourth, unlike in previous CG models, there is–by design–no microtubule growth or shortening in our model. The variability in protofilament lengths during the simulation is neglected in order to keep the modeling results statistically tractable. Therefore, in most cases, we can only make qualitative comparisons between cryoET and simulation, particularly for estimating the plausible range of lateral interaction energies *U_lat_* for GTP- and GDP-bound microtubule ends. Nevertheless, we believe *U_lat_* = 30–50 kJ/mol is a reasonable estimate because substantially lower or higher values yield no clusters or prevent microtubule flaring, respectively. Many of these limitations will be overcome in future by more elaborate CG mappings, parameterization schemes, and model generalizations as well as by more exhaustive atomistic MD simulations.

It is noteworthy that the excellent agreement between our simulations and experimental data (Fig. 4 *C* and *D*) also results from an improved signal-to-noise ratio provided by Cryo-CARE denoising (55). This procedure has allowed us to obtain higher resolution in 3D compared to previous studies (37–39). However, other limitations imposed by cryoET of flexible protofilaments remain. For example, missing wedge artifacts can result in undersampling of clustered protofilaments that are located in unfavorable orientations and can only be overcome by dual-axis tomography (82). Fully automated segmentation and tracing of denoised volumes may be necessary to increase the reliability and throughput of our method in the future.

Several important questions and concerns remain to be addressed. Although our simulation and cryoET data lead to the unexpected finding that it is mainly the conformation and stability of protofilament clusters—and not the overall shape—that determine the polymerization state of a microtubule end, we have not yet determined its critical structural ensemble for which the probabilities of growth and shortening are equal. Microtubule “aging”—the increase of the catastrophe probability with time—is another interesting but mechanistically not yet understood phenomenon, and it is currently unclear whether it can be explained within the conformational selection model or, alternatively, is caused by other factors, e.g., accumulating lattice defects (82). Additionally, our CG model so far cannot describe mixed nucleotide lattices which are also difficult to resolve experimentally. We believe that accurate CG models accounting for kinetic transitions caused by GTP hydrolysis and guided by the computational and structural findings presented here will contribute to our understanding of the mechanisms of catastrophe and rescue. To answer these crucial questions, we need to unify high-resolution electron and optical microscopy and biochemistry with advanced computational approaches in an integrative structural biology framework.

## Materials and Methods

### Preparation of In Vitro Microtubule Samples for CryoET.

Microtubules were polymerized using purified porcine brain tubulin (Cytoskeleton Inc) using double-cycled GMPCPP-stabilized seeds as templates (see *SI Appendix* *f*or details). Samples with growing microtubules ends were made by polymerizing microtubules in the presence of seeds, 15 µM tubulin, 1 mM GTP, and 5 nm gold nanoparticles at 37 °C for 30 min in a dry bath. 3.5 µL of this mixture were added to a freshly glow-discharged lacey carbon grid (Agar Scientific) suspended in the chamber of a Leica EM GP2 plunge-freezer equilibrated at 37 °C and 99% relative humidity. After the addition of microtubules, the grid was blotted from the back side and immediately frozen in liquid ethane.

Samples with shortening microtubules were made with DIG-labeled seeds attached to the surface of either 1.2/1.3 Quantifoil grids or silanized holey silicon oxide grids (SPI Supplies) as described previously (73). Briefly, the grids were incubated with anti-DIG IgG, washed with MRB80, then incubated with DIG-labeled GMPCPP-stabilized microtubule seeds and suspended in the chamber of a Leica EM GP2 plunge-freezer equilibrated at 37 °C and 99% relative humidity. 3 µL of 20 µM tubulin in MRB80 supplemented with 1 mM GTP were added to the grid and incubated in the chamber for 7 min. After that, 30 µL of prewarmed MRB80 were added to the grid to induce microtubule depolymerization, which proceeded for 30 to 45 s. Dilution buffer was supplemented with 5 nm gold nanoparticles before addition to the grid. The majority of the buffer dripped off from the grid, leaving 3 to 4 µL that were blotted off from the back side. Immediately after the blotting, the grid was frozen in liquid ethane. All grids were stored in liquid nitrogen until further use.

### Data Acquisition and Image Processing.

Bidirectional tilt series were recorded ranging from 0° to ±60° with a tilt increment of 2°, the total electron dose of 100 e^–^/Å^2^ and the target defocus set to -4 µm (see *SI Appendix* *f*or details of data acquisition). Tomograms were reconstructed and denoised as described previously, (55, 73) using tomograms generated with even and odd frames after alignment with MotionCor2, (83) and tilt series alignment and back projection performed in IMOD (84). Further analysis was limited to microtubule plus-ends. Microtubule polarity was determined using visual inspection of moiré patterns of protofilaments after Fourier filtering (85). Since microtubules were polymerized from GMPCPP seeds, most of them contained 14 protofilaments, making this analysis unambiguous in the majority of cases. The polarity was confirmed by observing microtubule cross-sections after the denoising procedure and noting the direction of protofilament “wedges” (86).

Each protofilament at a plus-end was manually traced using 3dmod (84). This procedure for manually tracing microtubule protofilaments is well documented and established, and it has been thoroughly tested in the original works (37, 38, 87). We introduced one modification to this method, enabled by the increased signal-to-noise ratio thanks to Cryo-CARE denoising (55). Specifically, the accuracy of the segmentation was monitored by simultaneously visualizing the manually placed coordinates overlaid with the rendered denoised experimental density in the *isosurface* view. The Cryo-CARE-enhanced procedure has been further shown to produce accurate and reproducible results in more recent studies (49, 88, 89). In addition, we estimated the variability in our own manual annotations by retracing a randomly selected microtubule end multiple times, and we observed only minor inconsistencies (*SI Appendix*, Fig. S9).

### Structural Analysis of Protofilament Clusters in CryoET Reconstructions.

To filter out and analyze protofilament clusters, we calculated the overlap between the volumes occupied by neighboring protofilaments. We equidistantly distributed spheres along the protofilament traces with an increment of 0.1 nm and set the equilibrium distance between two protofilaments in a cluster and the sphere radius to 5.34 nm and 3.20 nm, respectively.

During the analysis, we observed that in some cases two neighboring protofilaments, which were close to each other both at their initial segments and the tips, diverged at the mid-segments, creating a “bulge.” Because protofilaments have high bending rigidity, these bulges are energetically highly unfavorable, suggesting that they resulted from manual tracing errors and/or suboptimal resolution, particularly in orientations affected by the missing wedge.

To address this issue, we introduced a weighting procedure that placed greater emphasis on the more informative tip regions and reduced the impact of the less informative microtubule shaft segments. The weights $w_{i}$ increased linearly from 0 to 1 along the length shared by the protofilaments. The total volume overlap between two neighboring protofilament traces was calculated as the sum of the weighted neighboring sphere overlaps ${w_{i}\cdot \Delta V}_{i}$ and normalized by the sum of the weighted maximum volume overlaps ${w_{i}\cdot \Delta V}_{i,max}$ (assuming ideally straight protofilaments):

[1] $$\Omega =\sum_{i}w_{i}\cdot \Delta V_{i}/\sum_{i}w_{i}\cdot \Delta V_{i,max},$$

where ${\Delta V}_{i}\leq {\Delta V}_{i,max}$. The threshold for the volume overlap in a protofilament cluster was chosen to be Ω=0.1 (see *SI Appendix*, Fig. S7). This value indicates that at least 50% of the weighted linear distances between protofilaments deviate by less than 20% from the ideal case. *SI Appendix*, Fig. S8 additionally illustrates the “bulge” artifact problem along with a few examples of real 3D traces featuring such “bulges”. *SI Appendix* also provides a discussion on potential systematic bias during our cluster analysis.

### Discrete Elastic Rod Model of the Microtubule Lattice.

We used a discrete elastic rod (DER) representation to model individual protofilaments (90–93). This discrete differential geometry approach is designed to handle arbitrary deformable configurations of elastic materials, diverse cross-sections, and dynamic complexities. Here, we applied it to model the microscopic microtubule end structure as a set of 14 coupled DERs (Fig. 1*C*).

Each configuration of a DER was represented by a centerline consisting of N+1 nodes $r^{i}$, where $i\in \bar{0,N}$, connected by N edges. Each edge j, where $j\in \bar{0,N-1}$, was associated with a material frame $\left\{t^{j},m_{1}^{j},m_{2}^{j}\right\}$ that formed a right-handed orthonormal triad, with the tangential vector $t^{j}$ being oriented along the edge. The material frame described the orientation of the rod and, together with the twist-free (Bishop) frame, (90) was used to define the DER twist. Stretching, bending, and twisting deformations along the DER were represented by (i) the edge vector lengths $e^{j}=∥e^{j}∥=∥r^{j}-r^{j-1}∥$, (ii) the discrete integrated curvature vectors ${\left(\kappa b\right)}^{i}$, where $\kappa^{i}$ is the discrete integrated curvature and $b^{i}$ is the discrete binormal vector and (iii) the discrete integrated twists $m^{i}=\theta^{i}-\theta^{i-1}$, where $\theta^{i}$ is the angle between the material and the Bishop frame. To calculate this twist, we used the reference frame as described previously (91). Notably, in the DER formalism, stretching and compression are the properties of edges (pairs of nodes), while bending and twisting are the properties of nodes (pairs of edges), except for the terminal nodes that cannot be assigned a curvature or twist. In addition, twisting and bending of only neighboring edges relative to one another are correlated, i.e. no long-range effects along the protofilament are assumed.

The potential function U describing the energetics of the microtubule end was composed of the elastic energy of protofilaments and the lateral and longitudinal interaction energies between neighboring protofilaments (Fig. 1*D*). Following the canonical DER approach, the elastic energy was further composed of the stretching $U_{s}$, twisting $U_{t}$ and bending $U_{b}$ energies. An additional potential $U_{\mathrm{tb}}$ was introduced to explicitly account for the positive linear correlation between bending and twisting as this correlation was observed in our previous atomistic simulations of tubulin dimers and protofilaments (42, 56). All of these potentials were harmonic with respect to the elementary deformations, and their full mathematical expressions are given in *SI Appendix*, Table S1.

By definition, forces in the DER formalism act only on nodes and edge twist angles (i.e. the canonical coordinates), whereas a dimer in our system was described by 3 consecutive nodes in the DER (Fig. 1*C*). Consequently, the lateral interaction between two dimers in neighboring protofilaments was modeled as a 3-node interaction, with each pair of interacting nodes contributing one third. To this end, 2 virtual sites were introduced for every node in the protofilament located at distances -r and +r on the axis defined by $m_{2}^{j}$ of the associated edge (Fig. 1*D*), and the interaction between the virtual sites was modeled using a Morse potential. To account for volume exclusion, an additional repulsive potential between laterally neighboring nodes was introduced that was modeled using the repulsive part of a Lennard-Jones potential. Finally, we modeled the longitudinal interaction between two consecutive tubulin dimers in a protofilament by replacing the harmonic potential describing the stretching/compression of the edge corresponding to α-tubulin with another Morse potential (Fig. 1*D*). The full mathematical expressions for the lateral $U_{\mathrm{lat}}$ and longitudinal $U_{\mathrm{long}}$ interaction energies are given in *SI Appendix*, Table S1.

*SI Appendix* provides a more detailed description of the simulation framework, including Brownian Dynamics simulations, mapping, and parameterization of the coarse-grained microtubule end model (*SI Appendix*, Table S2) and all-atom free energy calculations for the longitudinal dimer–dimer bond.

## Supplementary Material

Appendix 01 (PDF)

Movie S1. Movie showing the dynamic cluster formation and dissociation at a GTP-microtubule end. *L_PF_* = 6 dimers and *U_lat_* = 40 kJ/mol. The duration of the simulation is 200 μs sampled with a time step of 250 ns.

Movie S2. Movie showing the dynamic cluster formation and dissociation at a GDP-microtubule end. *L_PF_* = 6 dimers and *U_lat_* = 40 kJ/mol. The duration of the simulation is 200 μs sampled with a time step of 250 ns.

## Data Availability

All MD simulations were done using GROMACS 2023 (94). All postprocessing calculations and data analyses were done with GROMACS internal tools, Python 3.9 (95), Numpy v1.26 (96), and SciPy v1.11 (97). Graphs were produced using Matplotlib v3.8.2 (98) and Seaborn v0.13 (99). All structure and cryoET density manipulations to produce images in the figures were performed using Chimera v1.17 (100) or Visual Molecular Dynamics (VMD) v1.9.3 (101). The VMD software was further used for visualization of microtubule end structures. All CG simulations of microtubule ends and protofilament clusters were performed using a custom Python code accelerated with Numba (https://github.com/moozzz/der_simulator_MT) (102). The full tomograms containing the growing and shortening plus-ends shown in Fig. 4*A* were deposited to EMDB (EMD-52025 and EMD-52026), and the repositories containing all raw data related to these EMDB entries were created (EMPIAR-12554 and EMPIAR-12555). The full set of denoised subtomograms containing microtubule plus-ends and results of their manual segmentation used in our analyses as well as the Python script for the cluster analysis shown in Fig. 4*C* were deposited to GRO.data, the Göttingen Research Online Database (https://data.goettingen-research-online.de/dataverse/pnas2025_pf_clusters_kalutskii_et_al). All other data are included in the manuscript and/or supporting information.
