# Supplementary material for: Microtubule dynamics are defined by conformations and stability of clustered protofilaments
Source: Proc Natl Acad Sci U S A. 2025 May 29;122(22):e2424263122. doi: 10.1073/pnas.2424263122 (PMC12146719; doi:10.1073/pnas.2424263122)
Supplement: Supplementary file 1 — Appendix 01 (PDF) [file pnas.2424263122.sapp.pdf]

PNAS

Supporting Information for

Microtubule dynamics are defined by conformations and stability of clustered protofilaments.

Maksim Kalutskii[a], Helmut Grubmüller[a], Vladimir A. Volkov[b,1], Maxim Igaev[a,c,1]

[a]Department of Theoretical and Computational Biophysics, Max Planck Institute for Multidisciplinary Sciences, Am Fassberg 11, D-37077 Göttingen, Germany

[b]Centre for Molecular Cell Biology, School of Biological and Behavioural Sciences, Queen Mary University of London, Mile End Road, London E1 4NS, United Kingdom

[c]Division of Computational Biology, School of Life Sciences, University of Dundee, Dow Street, Dundee DD1 5EH, United Kingdom

[1]To whom the correspondence may be addressed.

Email: migaev001@dundee.ac.uk and v.volkov@qmul.ac.uk

This PDF file includes:

SI Text
Figures S1 to S9 with captions
Tables S1 to S2
Legends for Movies S1 to S2
SI References

Other supporting materials for this manuscript include the following:

Movies S1 to S2
Figure S6 in high resolution in SVG format

Preparation of GMPCPP-stabilized microtubule seeds.
First, we prepared stabilized microtubule seeds by polymerizing 25 µM tubulin (with or without addition of 40% digoxigenin (DIG)-labeled tubulin) in the presence of 1 mM GMPCPP, a slowly hydrolyzable GTP analog. After 30 min of polymerization at 37°C, seeds were sedimented in a Beckman Airfuge, resuspended on ice in MRB80 (80 mM K-Pipes pH 6.9 with 4 mM MgCl2 and 1 mM EGTA). After 20 min of depolymerization on ice, seeds were re-polymerized in the presence of freshly added 1 mM GMPCPP, sedimented again, resuspended in MRB80 with 10% glycerol at room temperature, aliquoted and snap-frozen in liquid nitrogen.

Tomography data acquisition.
Growing microtubules (three separate samples) were imaged using a JEM-3200FSC electron microscope (JEOL) equipped with a K2 Summit direct electron detector (Gatan) and an in-column energy filter operated in zero-loss imaging mode with a 30-eV slit width. Images were recorded at 300 kV with a nominal magnification of 10,000x, resulting in the pixel size of 3.668 Å at the specimen level. Automated image acquisition was performed using SerialEM software.[1] A subset of growing microtubules (one additional sample) was imaged using a Titan Krios microscope equipped with a Gatan K3 electron detector at the Netherlands Center for Electron Nanoscopy (NeCEN, Leiden, Netherlands), at 300 kV with a nominal magnification of 26000x, resulting in the pixel size of 3.28 Å at the specimen level. Automated image acquisition was performed using SerialEM. Shortening microtubules (two separate samples) were imaged using a Titan Krios microscope (FEI) equipped with a Gatan K2 electron detector (NeCEN). Automated image acquisition was performed using Tomography software (Thermo Fisher). Images were recorded at 300 kV with a nominal magnification of 33,000x, resulting in the pixel size of 4.24 Å at the specimen level. Energy filtering for all data collected at NeCEN was performed at post-processing.

Considerations regarding human bias during manual segmentation.
Human bias is an inherent concern in any manual tracing technique. However, "blinding" the individual doing the manual tracing and cluster analysis with respect to the type of the microtubule dataset (growing or shortening) was challenging, because the two datasets were very different. The dataset with shortening microtubules contained a visibly large amount of "ram's horn" tubulin oligomers in solution that already detached from shortening microtubule ends and densely filled the space between them. In contrast, the dataset with growing microtubules only contained tubulin dimers and very short oligomers, making it quite obvious which dataset was being analyzed. The datasets we report here were collected between 2019 and 2021 – with a vast majority of manual annotations performed before the publication of our previous computational study[2] predicting the existence and functional importance of protofilament clusters. In addition, the manual tracing and the cluster analysis were performed independently by two different authors of this study (VAV and MK, respectively). Given the substantially increased signal-to-noise ratio due to the use of Cryo-CARE, we thus believe that the effect of human bias is minimized and does not affect the results and conclusions of this study.

Considerations regarding systematic bias during our cluster analysis.
We note that the primary determinant of cluster formation in our approach is sufficient sphere overlap. The weighting procedure does not affect this overlap, because it does not change the radius of the spheres placed along the protofilament traces. Instead, it modulates how much these local sphere overlaps ΔV_i contribute to the total overlap along the protofilaments. We apply it consistently to all pairs of neighboring traces in the 3D reconstructions, be they derived from a noisier or higher-resolution region. Therefore, our cluster analysis is unlikely to be biased towards the detection of clusters in noisier tomograms. In addition, we only consider two protofilaments as part of one cluster if they interact along their entire lengths. Two protofilaments that cluster near the microtubule shaft but separate closer to their tips are classified as single (see Fig. S8). As a result, our cluster analysis may slightly underestimate the number and size of clusters. We expect that, if the resolution of our tomograms was higher and equally high in all regions (no missing wedge), the number of identified clusters would either remain the same or increase.

Brownian Dynamics simulations.

To quantify the time evolution of the microtubule end, we integrated the overdamped Langevin equations of motion for the node positions $\boldsymbol{r}^i$ and the edge twist angles θ^j in every protofilament k simultaneously:

$$\boldsymbol{r}_{n+1}^{ik} = \boldsymbol{r}_n^{ik} - \frac{D_r \Delta t}{k_B T} \frac{\partial U}{\partial \boldsymbol{r}_n^{ik}} + \sqrt{2 D_r \Delta t} G_r \tag{2}$$

$$\theta_{n+1}^{ik} = \theta_n^{ik} - \frac{D_\theta \Delta t}{k_B T} \frac{\partial U}{\partial \theta_n^{ik}} + \sqrt{2 D_\theta \Delta t} G_\theta \tag{3}$$

where Δt is the integration time between steps $n + 1$ and n, $U = U_s + U_t + U_b + U_{tb} + U_{lat} + U_{long}$ is the full potential function, D_r and D_θ are the translational and rotational diffusion constants for the nodes and twist angles, k_B is the Boltzmann constant, T is the temperature, and G_r and G_θ are Gaussian distributed random numbers with zero mean and unit variance. $D_r \Delta t$ and $D_\theta \Delta t$ were adjusted such as to roughly reproduce the relaxation timescales of both the single protofilaments and the entire microtubule from our previous simulations ($D_r \Delta t \approx 2 \times 10^{-4}\ \mathrm{nm}^2$ and $D_\theta \Delta t \approx 2\ \times 10^{-4}\ \mathrm{rad}^2$).[2]

Mapping and parametrization of the coarse-grained microtubule end model.

There are two distinct stages to constructing a CG model: mapping and parametrization. The mapping procedure defines the resolution of a CG model and how well it captures the properties of structure, mechanics and symmetry.[3] In our case, the task was to find a mapping of the DER's centerline to the atomistic structure of a protofilament. To decouple stretching/compression deformations from bending deformations, this centerline should pass through groups of atoms in the protofilament structure such that the mutual distances between these groups do not change during protofilament bending, i.e. through "hinges" connecting tubulin monomers around which dimers twist and bend. Early structural studies identified helices H8 and H11' as key interaction sites between the monomers in a tubulin dimer and the dimers in a protofilament.[4,5] It was later shown computationally that most protofilament bending and twisting was enabled by these small and robust "hinges".[2,6,7]

We used the residues βH8:249–264 and αH11':405–411 and αH8:251–266 and βH11':395–401 to define the nodes $\boldsymbol{r}^{ik}$ corresponding to intra- and inter-dimer interfaces in the DER model, respectively. We further specified the material frames $\{\boldsymbol{t}^{jk}, \boldsymbol{m}_1^{jk}, \boldsymbol{m}_2^{jk}\}$ for the edges to complete the mapping. To this end, we drew an imaginary line that connected each edge's center of mass (COM) with the main microtubule axis and that was orthogonal to that edge. We then selected a group of atoms within a sphere of radius 0.25 nm located 2 nm away from the edge's COM on this line. We finally constructed a monomer vector between the edge's COM and this group of atoms for every tubulin monomer in the system. Finally, the material vectors $\boldsymbol{m}_1^{jk}$ and $\boldsymbol{t}^{jk}$ were defined as the normalized monomer and edge vectors, respectively. The material vectors $\boldsymbol{m}_2^{jk}$ were naturally defined to form a right-handed triad with $\boldsymbol{m}_1^{jk}$ and $\boldsymbol{t}^{jk}$.

Methods for the parametrization stage are diverse, and the models they produce can have distinct levels of accuracy.[3] In our case, the optimization task for a single protofilament was to obtain CG parameters that, given the above mapping, best reproduce the global essential dynamics of bending-torsional fluctuations of protofilaments.[2] To this end, we used fuzzy self-tuning particle swarm optimization (FST-PSO)[8,9] to minimize a custom-made objective function employing (i) Earth mover's distance (EMD)[10] as a metric to compare all-atom MD and CG distributions of local deformations and (ii) Principal Component Analysis (PCA)[11] as a metric to compare all-atom MD and CG distributions of the global essential dynamics (see Fig. S1C). The objective function was as follows:

$$O = \sqrt{\sum_{j=0}^{N-1} \delta_{s,j}^2} + \sqrt{\sum_{i=1}^{N-1} \delta_{b_1,i}^2} + \sqrt{\sum_{i=1}^{N-1} \delta_{tb_2,i}^2} + \sqrt{\sum_{p=1}^{2} \left(\lambda_p^{MD} - \lambda_p^{CG}\right)^2} + \sqrt{\sum_{p=1}^{2} \left(1 - \boldsymbol{v}_p^{MD} \cdot \boldsymbol{v}_p^{CG}\right)} \qquad (4)$$

where $\delta_{s,j}$ is the EMD between the all-atom MD and CG distribution of the j-th edge vector length, $\delta_{b_1,j}$ is the EMD between the all-atom MD and CG distribution of the first principal curvature for th i-th non-terminal node, $\delta_{tb_2,j}$ is the EMD between the all-atom MD and CG joint 2D distribution of the second principal curvature and twist for the i-th non-terminal node, and λ_p and $\boldsymbol{v}_p$ are the eigenvalues and the eigenvectors of the first 2 PCA components describing the protofilament dynamics in all-atom MD or CG representation.[2] The first and second principal curvatures are projections of the discrete integrated curvature vectors $(\kappa\boldsymbol{b})^i$ onto the material frames associated with the i-th node (see Jawed et al.[12] for the exact definitions) and describe radial and tangential bending of the DER, respectively.

There were a total of 18 parameters describing the deformation of a PF: $E_s^{\alpha,\beta}$, $E_{b_1}^{in,out}$, $E_{b_2}^{in,out}$, $E_t^{in,out}$, $E_{tb_2}^{in,out}$ (stretching, bending, twisting and twist-bending moduli, respectively) and $e_0^{\alpha,\beta}$, $\kappa_{1,0}^{in,out}$, $\kappa_{2,0}^{in,out}$, $m_0^{in,out}$ (equilibrium edge lengths, curvatures and twists, respectively), where the indices α and β denote edges corresponding to α- and β-tubulin and the indices in and out denote nodes corresponding to intra- and inter-dimer interfaces, respectively. Since the bending, twisting and twist-bending coupling terms build a positive definite quadratic form, their rigidity parameters must additionally satisfy the condition $E_t E_{b_2} - E_{tb_2}^2 \geq 0$ for every node. To simplify the problem, $e_0^{\alpha,\beta}$, $\kappa_{1,0}^{in,out}$, $\kappa_{2,0}^{in,out}$ and $m_0^{in,out}$ were not optimized explicitly; instead, they were set to the mean values obtained from the all-atom MD simulations converted to the DER representation. To achieve fast convergence and avoid sampling local minima of the 10-dimensional rigidity parameter space, we set up and ran 2200 independent FST-PSO optimizations, each starting from a random vector. The search space was limited to a physically reasonable range of $[10^2,\ 4 \times 10^4]$ for each parameter (regardless of the units), and the optimization was terminated when the objective function did not decrease for 1000 consecutive steps. For each of the optimized parameters, we collected a distribution of the 2200 FST-PSO solutions and calculated the mean value, which was then chosen to be the final optimization result (see Fig. S1D and Table S2). We finally obtained 2 separate sets of the optimized parameters for both GTP and GDP systems to account for the effect of the nucleotide state.

As specified above, the lateral and longitudinal dimer-dimer interaction energies were approximated with Morse potentials, with a repulsive Lennard-Jones component added to the lateral energy. The Morse potentials contained a total of 6 parameters: the interaction energies $U_{lat,long}^0$, the scaling terms $a_{lat,long}$ and the equilibrium distances $r_{lat,long}^0$, where the indices lat and $long$ denote lateral and longitudinal interaction interfaces, respectively. To simplify the problem, the repulsive Lennard-Jones constant σ was fixed and set to 5.34 nm, the COM-COM distance between neighboring dimers in a microtubule lattice. The equilibrium distances $r_{lat,long}^0$ were not optimized explicitly; instead, they were fixed and set to the values obtained from the all-atom free energy calculations. The optimization was performed only for parameters $U_{lat,long}^0$ and $a_{lat,long}$ as described above for the DER using a simplified objective function:

$$O = \delta_{lat} + \delta_{long} \qquad (5)$$

where δ_{lat} and δ_{long} are the EMDs between the all-atom MD and CG distributions of the COM-COM distance for a pair of laterally and longitudinally coupled dimers or monomers, respectively (see Table S2). Importantly, we later used the lateral interaction energy parameter U_{lat}^0 as an external free parameter in our study to manually control the "tug-of-war" balance (Fig. 1A), and a

dimensionless scaling factor was introduced into the system's potential function to control its magnitude.

To optimize the lateral parameters, we used the free energy calculations from our previous study (see Fig. S1E),[2] while we performed new free energy calculations for the longitudinal parameters (see Fig. S1F). We finally obtained 4 separate sets of the optimized parameters for the lateral interaction for both GTP and GDP systems and both homotypic and seam interfaces as well as 2 separate parameter sets for the longitudinal interaction for both GTP and GDP systems. In total, the atomistic datasets used to parametrize our CG model of the microtubule end comprised approximately 400 μs of cumulative sampling.

All-atom free energy calculations for the longitudinal dimer-dimer bond.
GTP- and GDP-tubulin oligomer systems composed of two longitudinally coupled dimers were prepared, solvated, neutralized with 150 mM KCl and equilibrated as described previously.[2] We employed the umbrella sampling approach[13] in conjunction with the weighted histogram analysis method (WHAM).[14] We first defined the COM-COM distance between α- and β-tubulin belonging to the inter-dimer interface as the reaction coordinate. The biasing potential was tuned to be 4000 $kJ/mol/nm^2$. To cover the full range of inter-dimer interactions, the COM-COM distance range between 4.2 and 6.5 nm was split into windows, each being separated by 0.05 nm. This partitioning of the reaction coordinate space yielded sufficient overlap between neighboring windows in the absolute majority of cases. The equilibration run was used for seeding the umbrella simulations, where each seed was separated from all the others by at least 50 ns in time. Seeding structures for those windows that were not initially covered by the equilibration simulation of the oligomer systems were derived from neighboring windows located 0.05 nm away in the reaction coordinate space. Each window was then simulated for 500 ns. We calculated the free energy profiles and their uncertainties using WHAM and Bayesian bootstrapping of the complete histograms scaled by inefficiency factors.[15]

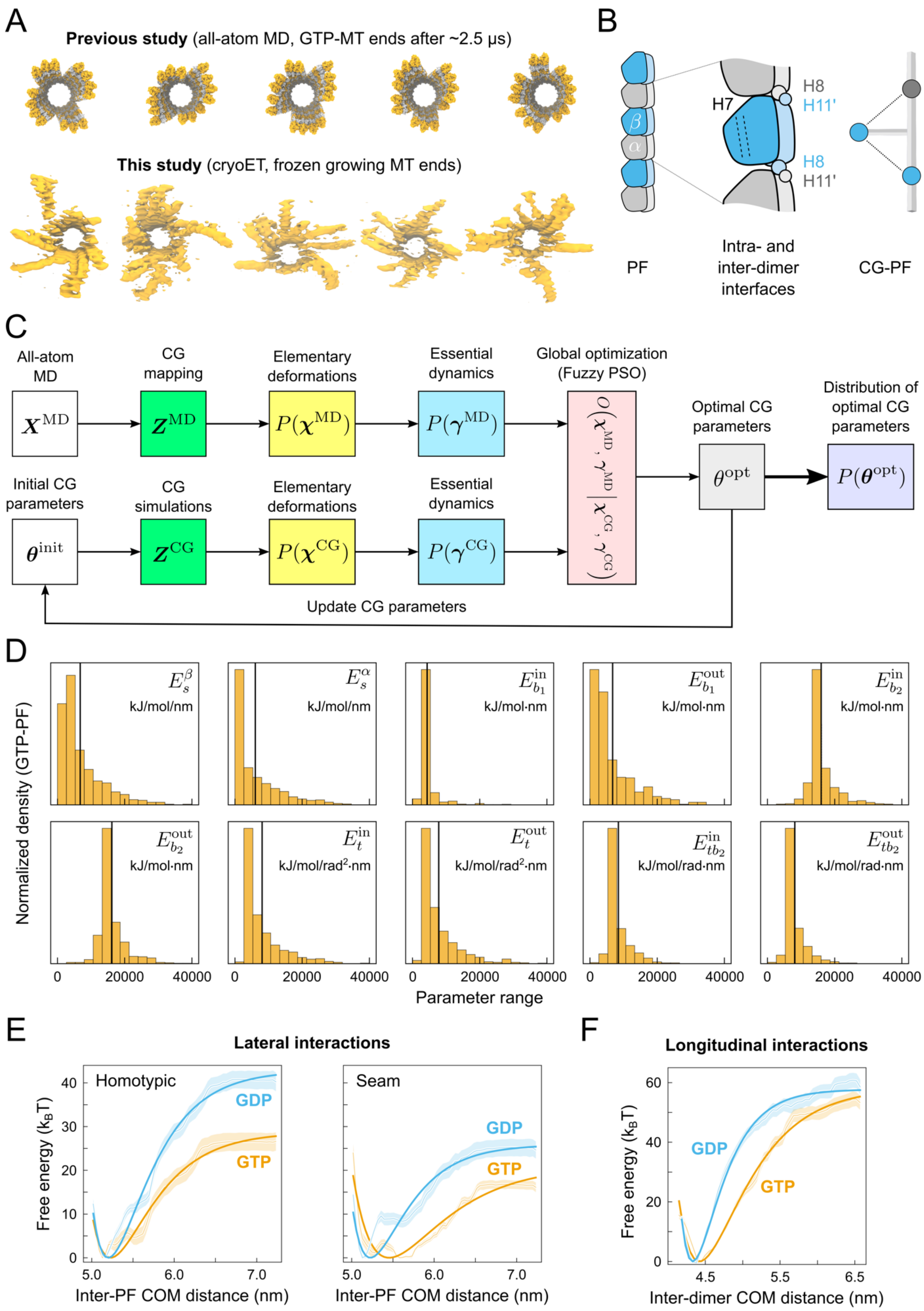
A
Previous study (all-atom MD, GTP-MT ends after ~2.5 μs)
This study (cryoET, frozen growing MT ends)
B
H8
H11'
H7
H8
H11'
PF
Intra- and inter-dimer interfaces
CG-PF
C
All-atom MD
CG mapping
Elementary deformations
Essential dynamics
Global optimization (Fuzzy PSO)
Initial CG parameters
CG simulations
Elementary deformations
Essential dynamics
Optimal CG parameters
Distribution of optimal CG parameters
Update CG parameters
D
Normalized density (GTP-PF)
kJ/mol/nm
kJ/mol/nm
kJ/mol·nm
kJ/mol·nm
kJ/mol·nm
kJ/mol·nm
kJ/mol/rad²·nm
kJ/mol/rad²·nm
kJ/mol/rad·nm
kJ/mol/rad·nm
0
20000
40000
Parameter range
E
Lateral interactions
Homotypic
GDP
GTP
Seam
GDP
GTP
Free energy (kBT)
Inter-PF COM distance (nm)
F
Longitudinal interactions
GDP
GTP
Free energy (kBT)
Inter-dimer COM distance (nm)

Figure S1. (A) Comparison of the simulated GTP-microtubule ends from our previous study[2] (top) with exemplary 3D rendered volumes of growing microtubule plus-ends obtained in this study (bottom). **(B)** Schematic illustration of the mapping defining the protofilament centerline. Helices H8 and H11' were used to define the DER nodes, while a group of atoms located within a sphere of radius 2 nm around helix H7 were used to define the material frame. **(C)** Flowchart diagram illustrating the optimization algorithm. **(D)** Distributions of parameters for the GTP-protofilament collected from 2200 independent FST-PSO optimizations (see Table S2 for the full list of parameters for both nucleotide states). The mean values are indicated with black vertical lines. **(E)** Free energy profiles of homotypic (left) and seam-like (right) tubulin–tubulin lateral interactions between two straight, infinitely long protofilaments as a function of the inter-protofilament COM distance.[2] Color coding for GTP and GDP as in Fig. 2. Solid lines indicate the Morse potentials in our CG model optimized against these atomistic data (see Table S2 for the full list of parameters for both nucleotide states). **(F)** Free energy profiles of tubulin-tubulin longitudinal interactions in a short protofilament consisting of two dimers. Color coding as in Fig. 2. Solid lines indicate the Morse potentials in our CG model optimized against these atomistic data (see Table S2 for the full list of parameters for both nucleotide states).

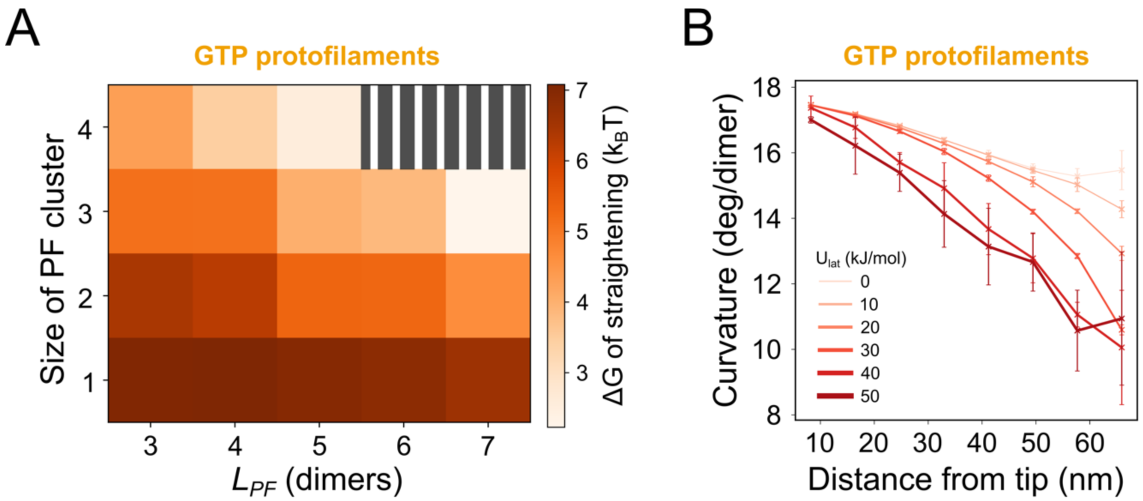

Figure S2. (A) Free energy required to straighten a GTP-cluster of size between 1 to 4 protofilaments and L_{PF} between 3 to 7 dimers. The shaded area indicates the parameters for which the formation of clusters was not possible due to excess strain and protofilament rupture. The same single-cluster setup was used as shown in Fig. S5. **(B)** Protofilament curvature as a function of the distance from the tip plotted for L_{PF} = 9 dimers and for U_{lat} between 0 and 50 kJ/mol. Error bars indicate the standard deviations within the simulated ensembles of 30 independent replicas each.

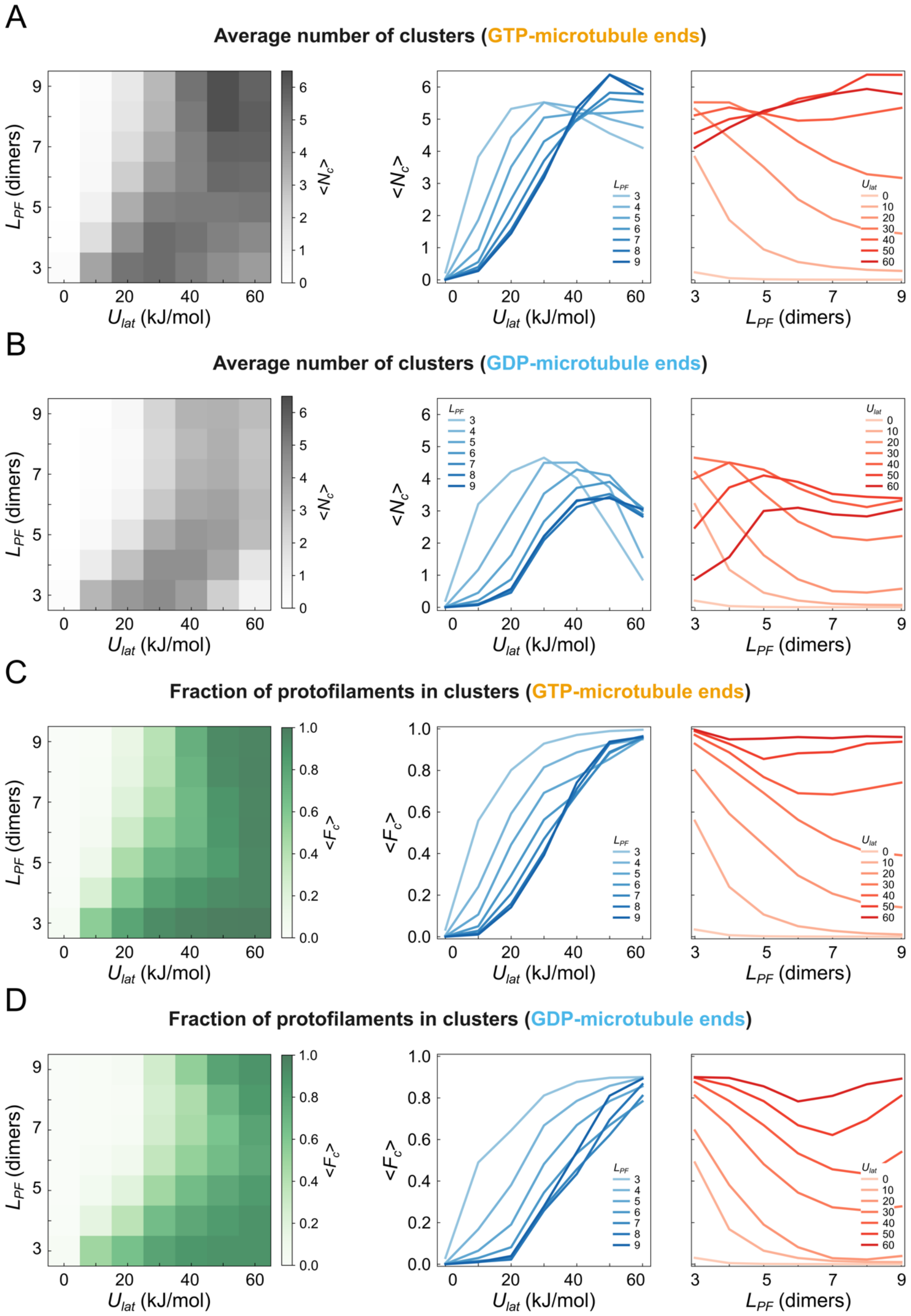
A
Average number of clusters (GTP-microtubule ends)
B
Average number of clusters (GDP-microtubule ends)
C
Fraction of protofilaments in clusters (GTP-microtubule ends)
D
Fraction of protofilaments in clusters (GDP-microtubule ends)
L_{PF} (dimers)
U_{lat} (kJ/mol)
$<N_c>$
$<F_c>$
L_{PF}
U_{lat}

Figure S3. Two-dimensional parametric diagrams of the average number of clusters (<N_c>) at **(A)** GTP- and **(B)** GDP-microtubule ends as well as the average fraction of protofilaments (<F_c>) in **(C)** GTP- and **(D)** GDP-clusters. Graphs on the left of each panel additionally show the same data as projections on either U_{lat} or L_{PF}.

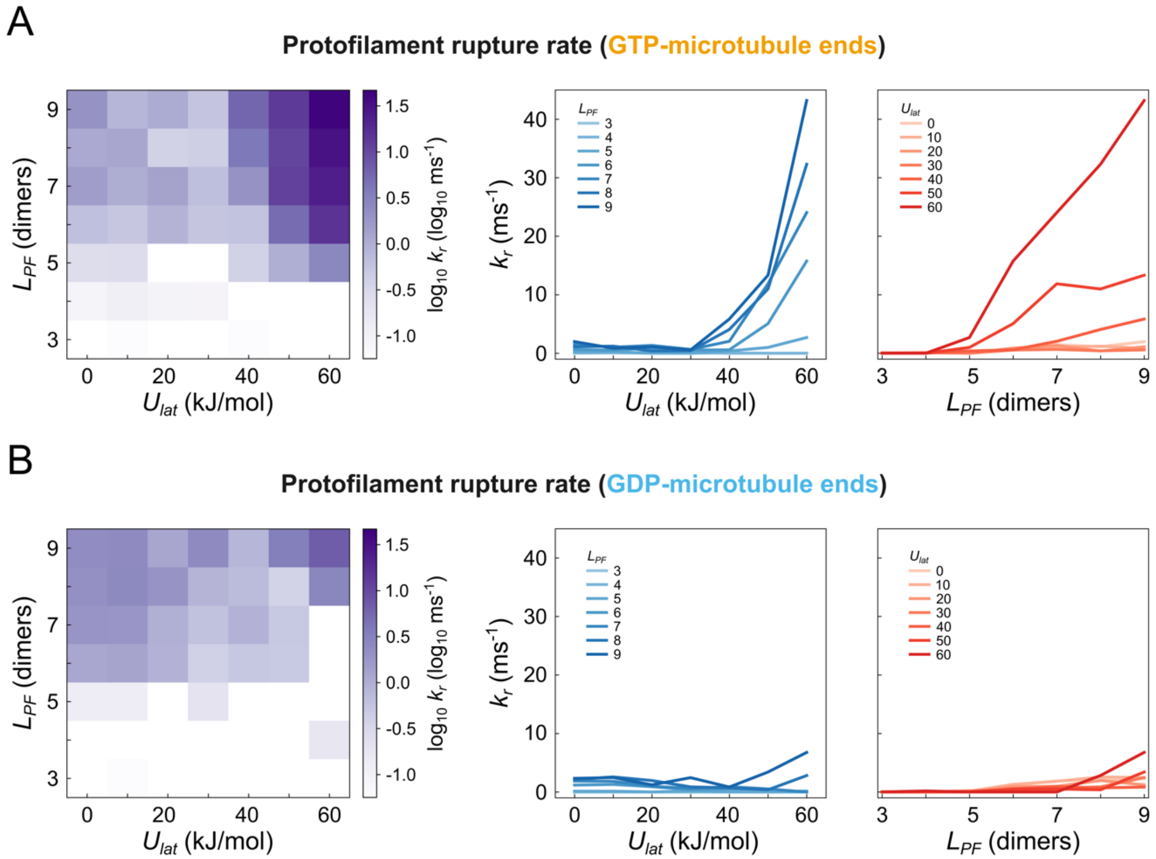

Figure S4. Two-dimensional parametric diagrams of the rupture rate (k_r) for **(A)** GTP- and **(B)** GDP-protofilaments as a function of lateral interaction strength (U_{lat}) and protofilament length (L_{PF}). Graphs on the left of each panel additionally show the same data as projections on either U_{lat} or L_{PF}. Note that the diagrams use a log-scale for clarity, while the additional graphs use a linear scale to plot the rupture rate.

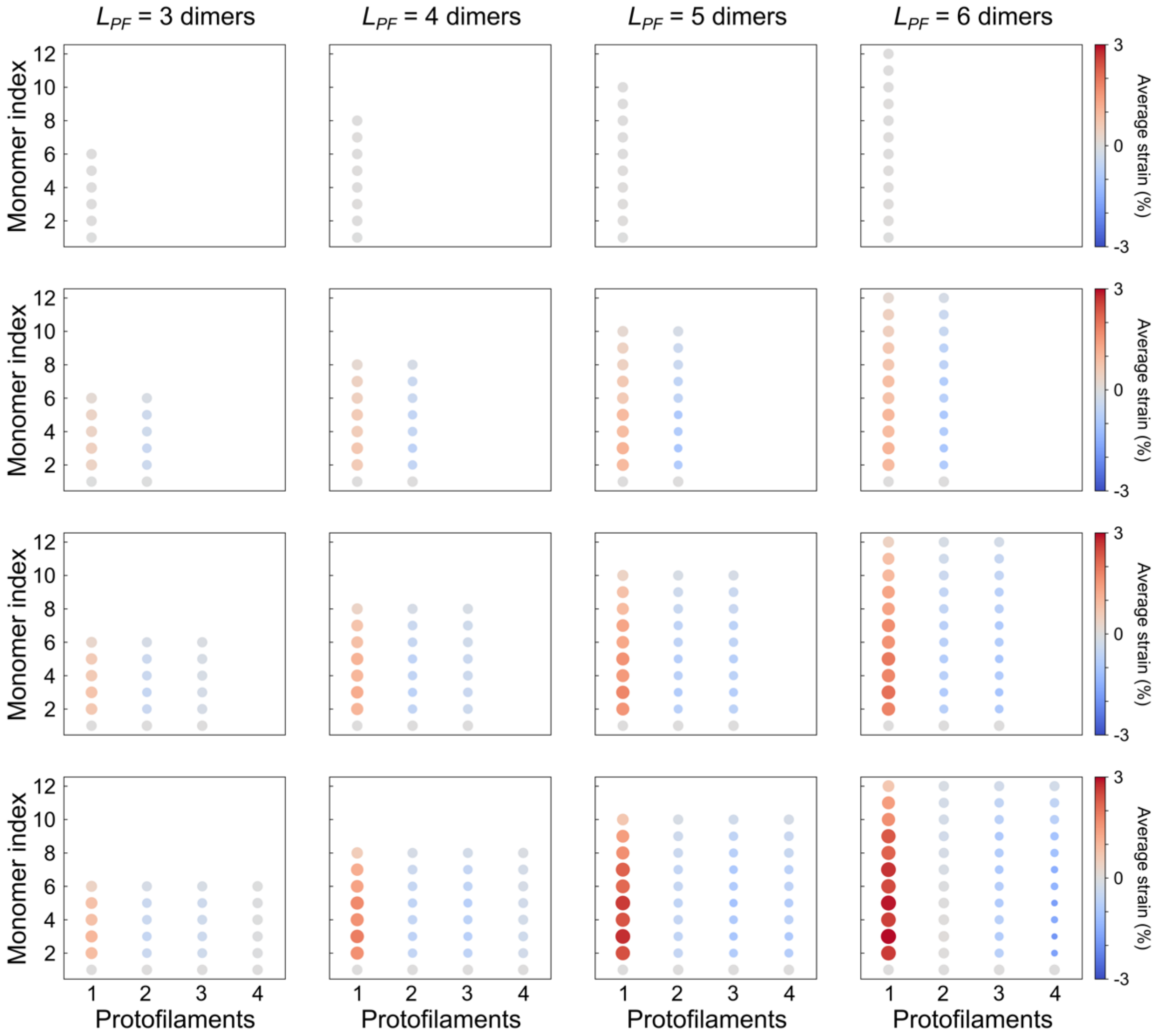

Figure S5. Two-dimensional parametric diagrams of the average relative strain along GTP-protofilaments in clusters of size 1 to 4 protofilaments (rows) and length 3 to 6 dimers (columns). Each circle corresponds to a tubulin monomer while its color and size denote the magnitude and the sign of strain, respectively. The lateral and longitudinal bonds were replaced with harmonic potentials to prevent dissociation. The same single-cluster setup was used as shown in Fig. S2 except that here, the lateral and longitudinal bonds were unbreakable.

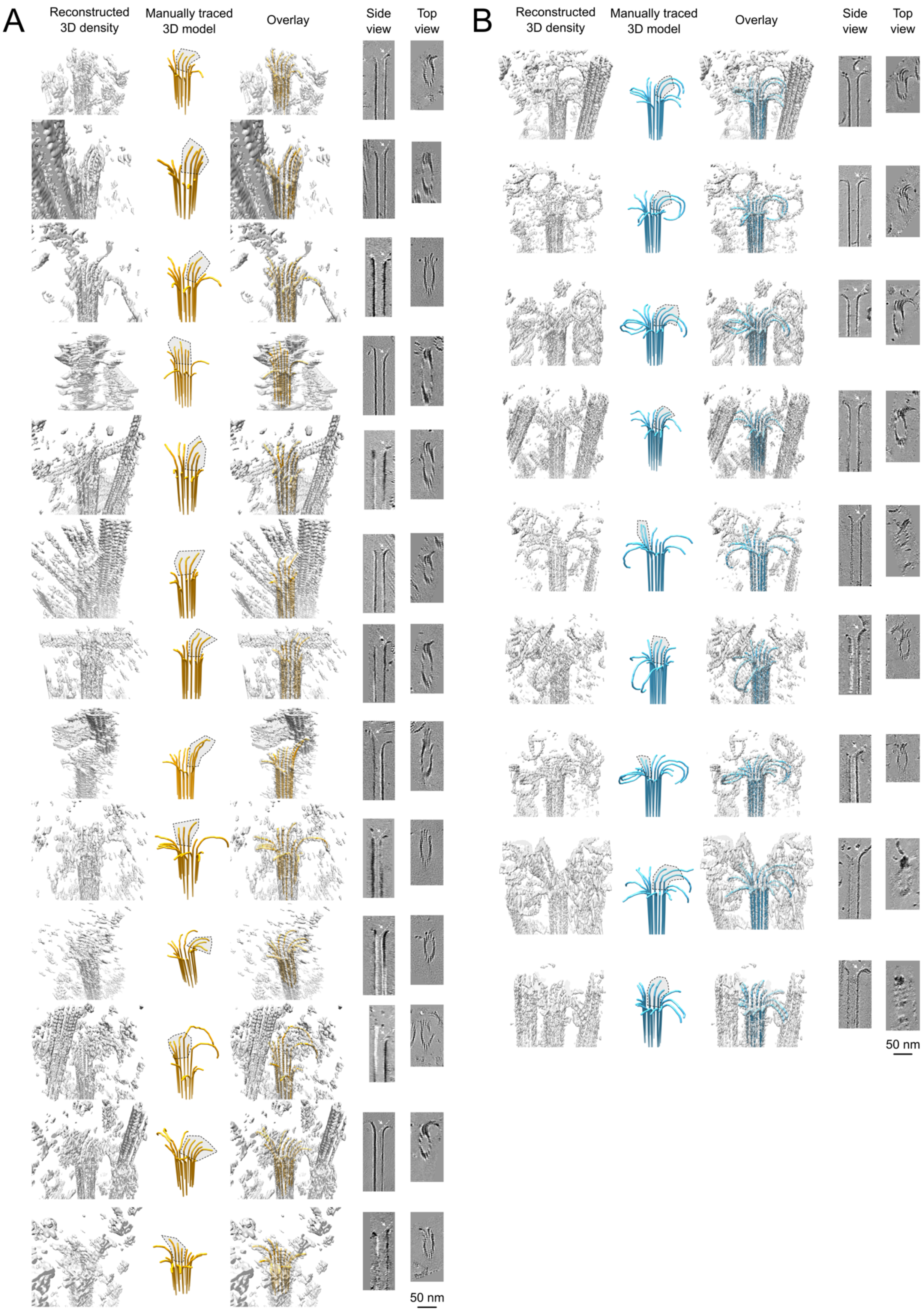
A
Reconstructed 3D density
Manually traced 3D model
Overlay
Side view
Top view
50 nm
B
Reconstructed 3D density
Manually traced 3D model
Overlay
Side view
Top view
50 nm

Figure S6. Examples of representative growing **(A)** and shortening **(B)** microtubule ends, in orientations similar to Fig 4A. Shown are from left to right the reconstructed 3D volumes, the manually traced 3D models, the overlay and the side and top views in the corresponding 2D tomograms. Gray areas highlight protofilament clusters. Scale bars are 50 nm.

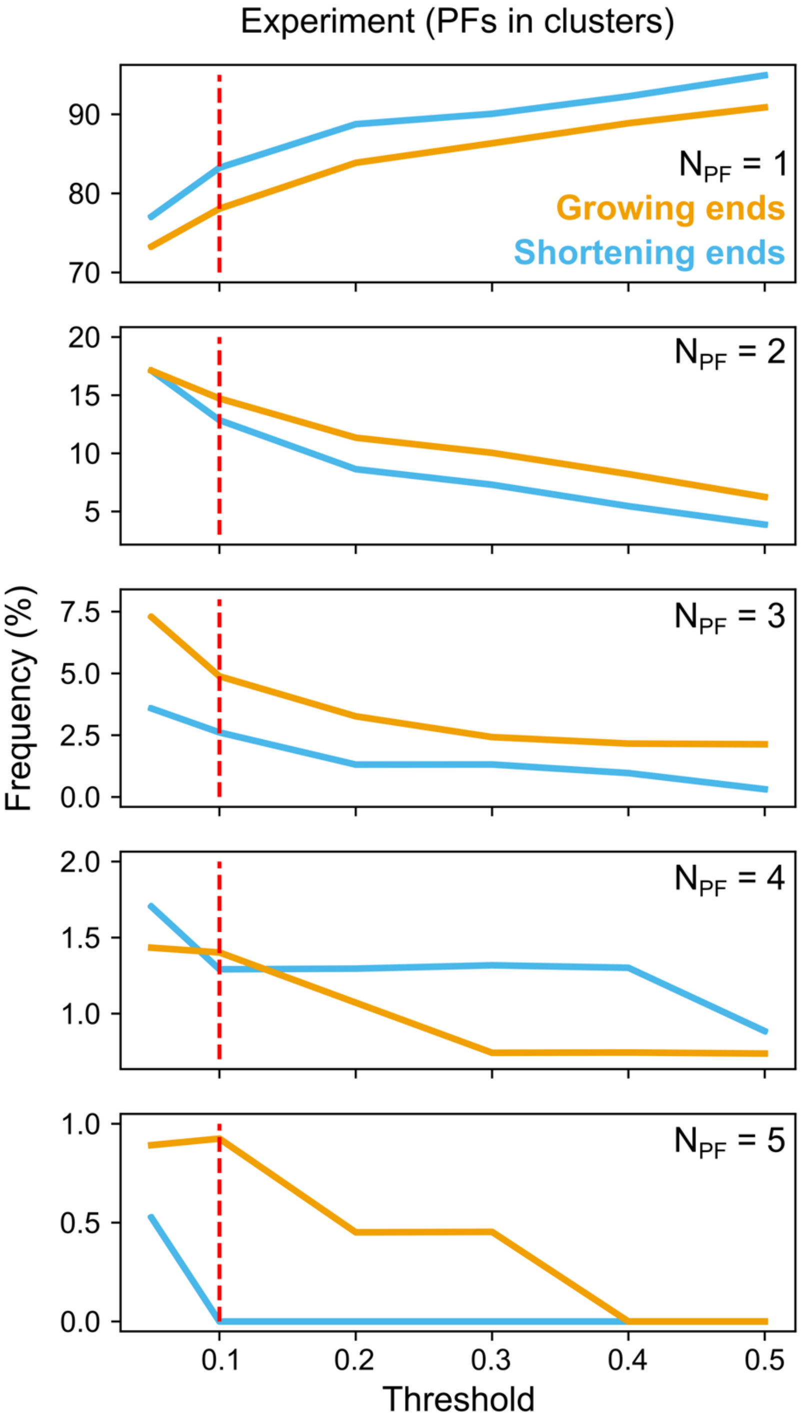

Figure S7. The relationship between the threshold in the overlap between neighboring protofilaments, used for defining a protofilament cluster, and the observed frequencies of clusters of various size in our dataset. The red line marks the chosen threshold of 10% used consistently throughout our study. Regardless of the chosen threshold, the quantitative difference between the detected clusters at growing and shrinking ends is preserved.

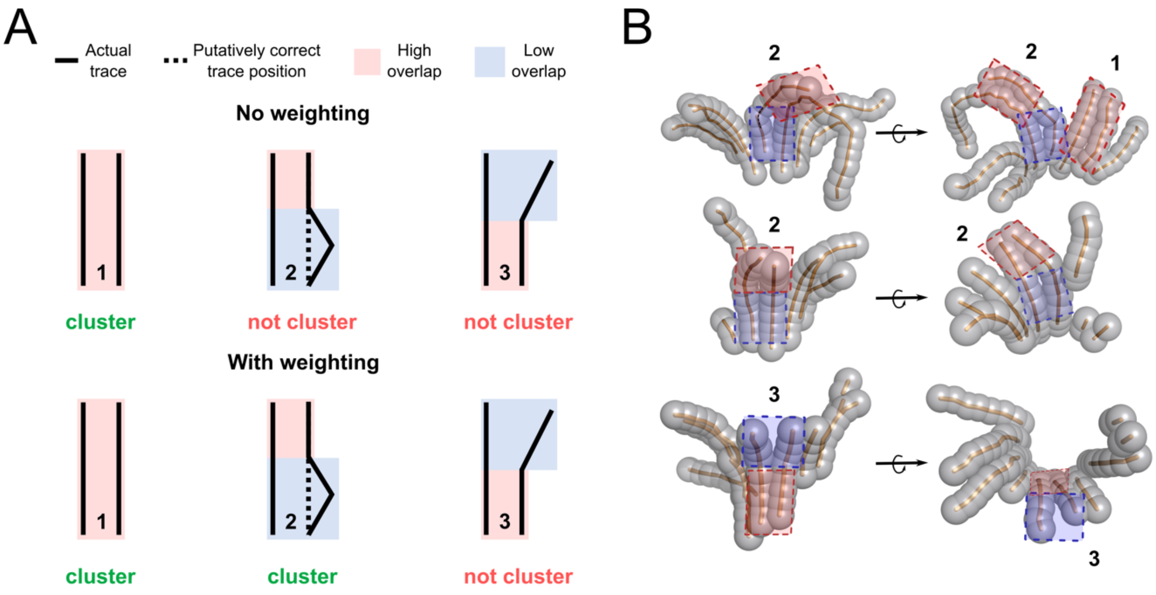

Figure S8. (A) Schematic illustration of a “normal” cluster (1), a “bulge” (2) and a pair of protofilaments splaying at a cluster’s tip (3). Our weighting procedure exclusively targets (2), while (1) and (3) remain unaltered upon the volume overlap weighting. Solid and dashed lines correspond to actual and putatively correct trace positions, respectively. Red and blue areas mark high- and low-volume overlaps, respectively. **(B)** Examples of each case in our traced datasets.

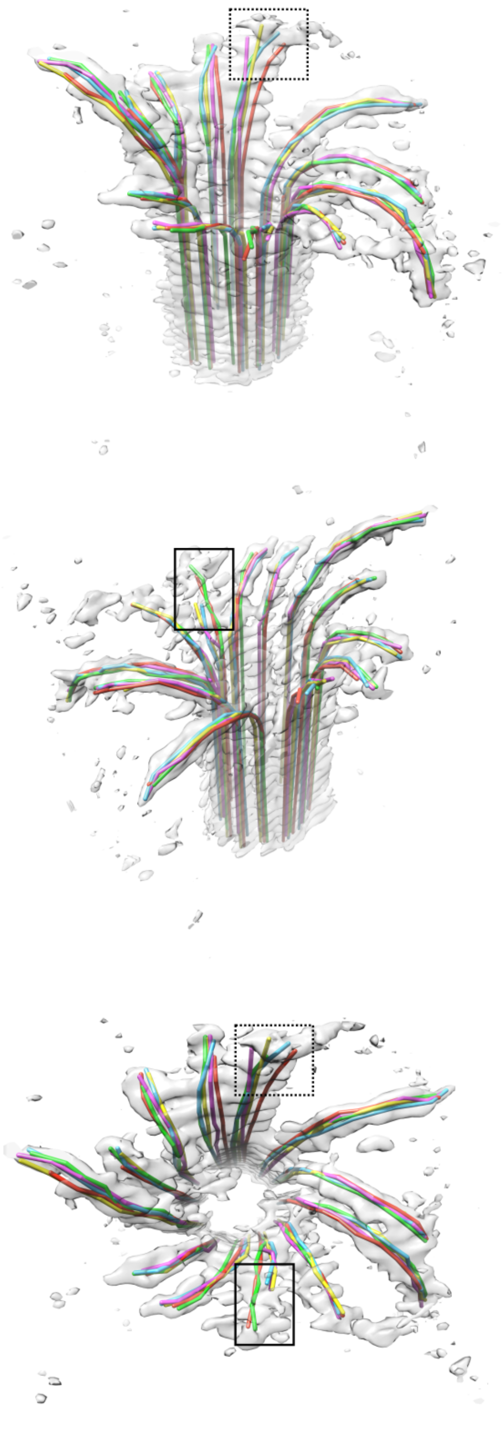

Figure S9. Assessment of the accuracy and reproducibility of our manual tracing procedure. Shown are randomly colored traces of the same microtubule end performed independently by the authors and overlaid with the corresponding microtubule end density. Solid and dashed rectangles indicate minor inconsistences in the different traces.

Table S1. Mathematical expressions for the elastic, lateral and longitudinal potential functions defined in Fig. 1D.[12] Also shown are expressions for the auxiliary quantities, i.e. relative extensions $\boldsymbol{\varepsilon^j}$, twists $\boldsymbol{m^i}$, principal curvatures $\boldsymbol{k_1^i}$ and $\boldsymbol{k_2^i}$. There are a total of 24 model parameters, from which only 14 parameters were optimized.

Potential	Expression	Schematic	Auxiliary quantities	Model parameters
Stretching	$U_s = \sum_{j=0}^{N-1} E_s^j \lVert\mathbf{e}_0^j\rVert {\varepsilon^j}^2$	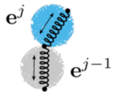	$\varepsilon^j = \frac{\lVert\mathbf{e}^j\rVert - \lVert\mathbf{e}_0^j\rVert}{\lVert\mathbf{e}_0^j\rVert}$<br>$E_s^j = E_s^\alpha$, if j is even<br>$E_s^j = E_s^\beta$, if j is odd<br>$\lVert\mathbf{e}_0^j\rVert = \lVert\mathbf{e}_0^\alpha\rVert$, if j is even<br>$\lVert\mathbf{e}_0^j\rVert = \lVert\mathbf{e}_0^\beta\rVert$, if j is odd	E_s^α E_s^β<br>$\lVert\mathbf{e}_0^\alpha\rVert$ $\lVert\mathbf{e}_0^\beta\rVert$
Twisting	$U_t = \sum_{i=1}^{N-1} \frac{E_t^i}{l_0^i}\left[m^i - m_0^i\right]^2$	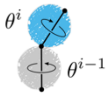	$m^i = \theta^i - \theta^{i-1}$<br>$l_0^i = \frac{1}{2}\left[\lVert\mathbf{e}_0^{i-1}\rVert + \lVert\mathbf{e}_0^i\rVert\right]$<br>$E_t^i = E_t^{in}$, if i is odd<br>$E_t^i = E_t^{out}$, if i is even<br>$m_0^i = m_0^{in}$, if i is odd<br>$m_0^i = m_0^{out}$, if i is even	E_t^{in} E_t^{out}<br>m_0^{in} m_0^{out}
Bending	$U_b = \sum_{i=1}^{N-1} \frac{E_{b_1}^i}{l^i}\left[\kappa_1^i - \kappa_{1,0}^i\right]^2 + \frac{E_{b_2}^i}{l^i}\left[\kappa_2^i - \kappa_{2,0}^i\right]^2$	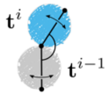	$\kappa_1^i = \frac{1}{2}\left[\mathbf{m}_2^{i-1} + \mathbf{m}_2^i\right]\cdot(\kappa\mathbf{b})^i$<br>$\kappa_2^i = -\frac{1}{2}\left[\mathbf{m}_1^{i-1} + \mathbf{m}_1^i\right]\cdot(\kappa\mathbf{b})^i$<br>$\mathbf{b}^i = \frac{\mathbf{t}^{i-1}\times\mathbf{t}^i}{\lVert\mathbf{t}^{i-1}\times\mathbf{t}^i\rVert}$<br>$\kappa^i = \mathrm{tg}\left(\frac{\mathbf{t}^{i-1}\wedge\mathbf{t}^i}{2}\right)$<br>$E_{b_{1,2}}^i = E_{b_{1,2}}^{in}$, if i is odd<br>$E_{b_{1,2}}^i = E_{b_{1,2}}^{out}$, if i is even<br>$\kappa_{1,2,0}^i = \kappa_{1,2,0}^{in}$, if i is odd<br>$\kappa_{1,2,0}^i = \kappa_{1,2,0}^{out}$, if i is even	$E_{b_{1,2}}^{in}$ $E_{b_{1,2}}^{out}$<br>$\kappa_{1,2,0}^{in}$ $\kappa_{1,2,0}^{out}$
Twist-bending coupling	$U_{tb} = \sum_{i=1}^{N-1} \frac{E_{tb_2}^i}{l_0^i}\left[m^i - m_0^i\right]\left[\kappa_2^i - \kappa_{2,0}^i\right]$	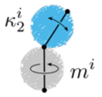	$E_{tb_2}^i = E_{tb_2}^{in}$, if i is odd<br>$E_{tb_2}^i = E_{tb_2}^{out}$, if i is even	$E_{tb_2}^{in}$ $E_{tb_2}^{out}$
Lateral interactions	$U_{lat} = \sum_{p,q}^{pairs} \frac{U_{lat}^0}{3}\left[1 - e^{-a_{lat}\left(r_{lat}^{pq} - r_{lat}^0\right)}\right]^2 + \frac{U_{LJ}^0}{3}\left[\frac{\sigma_{LJ}^0}{R_{lat}^{pq}}\right]^{12}$	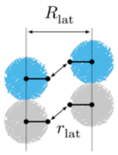		U_{lat}^0<br>a_{lat}<br>r_{lat}^0
Longitudinal interactions	$U_{long} = \sum_{p,q}^{pairs} U_{long}^0\left[1 - e^{-a_{long}\left(r_{long}^{pq} - r_{long}^0\right)}\right]^2$	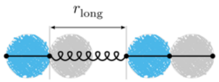		U_{long}^0<br>a_{long}<br>r_{long}^0

Table S2. Values of the model parameters used in this study. If longitudinal bonds are set to be breakable, the equilibrium edge lengths of α- and β-tubulin (top; marked with *) are replaced with the equilibrium bond distances of the Morse potentials (bottom; marked with *).

Parameter	Units	Optimized or fixed from MD?	Value (GTP)		Value (GDP)	
E_s^α	kJ/mol/nm	Optimized	6061.6		10492.3	
E_s^β	kJ/mol/nm	Optimized	6868.7		11397.3	
$\lVert\mathbf{e}_0^\alpha\rVert$	nm	Fixed	3.79*		3.78*	
$\lVert\mathbf{e}_0^\beta\rVert$	nm	Fixed	4.17		4.20	
E_t^{in}	kJ/mol/rad²·nm	Optimized	8036.8		12931.5	
E_t^{out}	kJ/mol/rad²·nm	Optimized	7841.7		12783.9	
m_0^{in}	rad	Fixed	-0.0147		-0.0103	
m_0^{out}	rad	Fixed	-0.0651		-0.1382	
$E_{b_1}^{\mathrm{in}}$	kJ/mol·nm	Optimized	4353.7		3250.9	
$E_{b_1}^{\mathrm{out}}$	kJ/mol·nm	Optimized	6724.3		13115.6	
$E_{b_2}^{\mathrm{in}}$	kJ/mol·nm	Optimized	16138.4		18479.7	
$E_{b_2}^{\mathrm{out}}$	kJ/mol·nm	Optimized	16170.8		17889.8	
$\kappa_{1,0}^{\mathrm{in}}$	–	Fixed	-0.0889		-0.0451	
$\kappa_{1,0}^{\mathrm{out}}$	–	Fixed	-0.3015		-0.2546	
$\kappa_{2,0}^{\mathrm{in}}$	–	Fixed	-0.0040		-0.0122	
$\kappa_{2,0}^{\mathrm{out}}$	–	Fixed	0.0621		0.0720	
$E_{tb_2}^{\mathrm{in}}$	kJ/mol/rad·nm	Optimized	8434.3		9665.8	
$E_{tb_2}^{\mathrm{out}}$	kJ/mol/rad·nm	Optimized	8276.8		9039.7	
			homo	*seam*	*homo*	*seam*
U_{lat}^0	kJ/mol	Optimized	28.7	21.0	42.9	25.9
a_{lat}	nm⁻¹	Optimized	2.06	1.53	2.16	2.36
r_{lat}^0	nm	Fixed	5.23	5.45	5.20	5.23
U_{long}^0	kJ/mol	Optimized	58.3		57.7	
a_{long}	nm⁻¹	Optimized	3.65		4.81	
r_{long}^0	nm	Fixed	4.42*		4.33*	

Movie S1 (separate file). Movie showing the dynamic cluster formation and dissociation at a GTP-microtubule end. L_{PF} = 6 dimers and U_{lat} = 40 kJ/mol. The duration of the simulation is 200 μs sampled with a time step of 250 ns.

Movie S2 (separate file). Movie showing the dynamic cluster formation and dissociation at a GDP-microtubule end. L_{PF} = 6 dimers and U_{lat} = 40 kJ/mol. The duration of the simulation is 200 μs sampled with a time step of 250 ns.

SI References

1. Mastronarde, D. N. Automated electron microscope tomography using robust prediction of specimen movements. *J. Struct. Biol.* **152**, 36–51 (2005).
2. Igaev, M. & Grubmüller, H. Bending-torsional elasticity and energetics of the plus-end microtubule tip. *Proc. Natl. Acad. Sci. U. S. A.* **119**, e2115516119 (2022).
3. Jin, J., Pak, A. J., Durumeric, A. E. P., Loose, T. D. & Voth, G. A. Bottom-up coarse-graining: Principles and perspectives. *J. Chem. Theory Comput.* **18**, 5759–5791 (2022).
4. Nogales, E., Wolf, S. G. & Downing, K. H. Structure of the alpha beta tubulin dimer by electron crystallography. *Nature* **391**, 199–203 (1998).
5. Löwe, J., Li, H., Downing, K. H. & Nogales, E. Refined structure of αβ-tubulin at 3.5 Å resolution. *J. Mol. Biol.* **313**, 1045–1057 (2001).
6. Grafmüller, A. & Voth, G. A. Intrinsic bending of microtubule protofilaments. *Structure* **19**, 409–417 (2011).
7. Fedorov, V. A. *et al.* Mechanical properties of tubulin intra- and inter-dimer interfaces and their implications for microtubule dynamic instability. *PLoS Comput. Biol.* **15**, e1007327 (2019).
8. Kennedy, J. & Eberhart, R. Particle swarm optimization. in *Proceedings of ICNN'95 - International Conference on Neural Networks* vol. 4 1942–1948 vol.4 (IEEE, 2002).
9. Nobile, M. S. *et al.* Fuzzy Self-Tuning PSO: A settings-free algorithm for global optimization. *Swarm Evol. Comput.* **39**, 70–85 (2018).
10. Rubner, Y. & Tomasi, C. The earth mover's distance. in *Perceptual Metrics for Image Database Navigation* 13–28 (Springer US, Boston, MA, 2001).
11. Amadei, A., Linssen, A. B. & Berendsen, H. J. Essential dynamics of proteins. *Proteins* **17**, 412–425 (1993).
12. Jawed, M. K., Novelia, A. & O'Reilly, O. M. *A Primer on the Kinematics of Discrete Elastic Rods*. (Springer International Publishing, Cham, Switzerland, 2018).
13. Torrie, G. M. & Valleau, J. P. Nonphysical sampling distributions in Monte Carlo free-energy estimation: Umbrella sampling. *J. Comput. Phys.* **23**, 187–199 (1977).

14. Kumar, S., Rosenberg, J. M., Bouzida, D., Swendsen, R. H. & Kollman, P. A. THE weighted histogram analysis method for free-energy calculations on biomolecules. I. The method. *J. Comput. Chem.* **13**, 1011–1021 (1992).
15. Hub, J. S., de Groot, B. L. & van der Spoel, D. G_wham—A free weighted histogram analysis implementation including robust error and autocorrelation estimates. *J. Chem. Theory Comput.* **6**, 3713–3720 (2010).
